# Development and field performance of nitrogen use efficient rice lines for Africa

**DOI:** 10.1111/pbi.12675

**Published:** 2017-01-25

**Authors:** Michael Gomez Selvaraj, Milton Orlando Valencia, Satoshi Ogawa, Yingzhi Lu, Liying Wu, Christopher Downs, Wayne Skinner, Zhongjin Lu, Jean C. Kridl, Manabu Ishitani, Jos van Boxtel

**Affiliations:** ^1^ International Center for Tropical Agriculture (CIAT) Cali Colombia; ^2^ Faculty of Agriculture Science National University of Colombia Palmira Colombia; ^3^ Japan Society for the Promotion of Science Postdoctoral Fellow Graduate School of Agricultural and Life Science The University of Tokyo Tokyo Japan; ^4^ Arcadia Biosciences Davis CA USA

**Keywords:** alanine aminotransferase, confined field trial, grain yield, nitrogen use efficiency, transgenic rice, transformation

## Abstract

Nitrogen (N) fertilizers are a major input cost in rice production, and its excess application leads to major environmental pollution. Development of rice varieties with improved nitrogen use efficiency (NUE) is essential for sustainable agriculture. Here, we report the results of field evaluations of marker‐free transgenic NERICA4 (New Rice for Africa 4) rice lines overexpressing barley alanine amino transferase (*HvAlaAT*) under the control of a rice stress‐inducible promoter (pOsAnt1). Field evaluations over three growing seasons and two rice growing ecologies (lowland and upland) revealed that grain yield of pOsAnt1:*HvAlaAT* transgenic events was significantly higher than sibling nulls and wild‐type controls under different N application rates. Our field results clearly demonstrated that this genetic modification can significantly increase the dry biomass and grain yield compared to controls under limited N supply. Increased yield in transgenic events was correlated with increased tiller and panicle number in the field, and evidence of early establishment of a vigorous root system in hydroponic growth. Our results suggest that expression of the *HvAlaAT* gene can improve NUE in rice without causing undesirable growth phenotypes. The NUE technology described in this article has the potential to significantly reduce the need for N fertilizer and simultaneously improve food security, augment farm economics and mitigate greenhouse gas emissions from the rice ecosystem.

## Introduction

According to FAO projections, by 2035, a 26% increase in rice production will be necessary to feed the growing population (Cassman *et al*., 2003; Seck *et al*., 2012). Farmers in sub‐Saharan Africa (SSA) produce about 12 million metric tons of rice annually, while importing another 12 million metric tons, which is valued at five billion dollars (FAO, 2016). Most of the rice in SSA is produced and consumed by small‐scale farmers who are often constrained by input costs, mainly fertilizer, and access to new technologies that could help them increase food production.

Nitrogen (N) is an essential nutrient and is often the most yield‐limiting nutrient in rice production around the world (Samonte *et al*., 2006). N fertilizer costs comprise an important fraction of total production costs in crops (Tirol‐Padre *et al*., 1996). Yet, estimates of the world nitrogen use efficiency (NUE) have been calculated to be as low as 33% for cereals (Raun and Johnson, 1999) and less than half of the applied N is recovered in the grain. The negative environmental effects of excess N are well documented (Glass, 2003; Raun and Johnson, 1999; Tilman, 1999). Therefore, there is an urgent need to increase NUE to mitigate the economic and environmental costs of rice production. Understanding and manipulating the mechanisms of crop N uptake and utilization at a molecular level may help to improve NUE in rice.

NUE crops were defined as crops that can more efficiently uptake, utilize and remobilize the N available to them (Han *et al*., 2015; McAllister *et al*., 2012). Two approaches have been used to increase NUE in crop plants. The first involves both conventional breeding and marker‐assisted selection in an attempt to identify the major QTLs involved (Gallais and Hirel, 2004; Hirel *et al*., 2001; Masclaux‐Daubresse *et al*., 2010). The second approach uses novel gene constructs designed to improve specific aspects of NUE (Good *et al*., 2004). Overexpression of key enzymes involved in N metabolism has been considered as a strategy to enhance NUE (Moose and Below, 2009). However, the results of such attempts in transgenic crops by ectopic regulation of these key enzymes (Anbessa and Juskiw, 2012; Good *et al*., 2004; Hirel *et al*., 2007; Kant *et al*., 2011), in most cases, did not yield significant improvements (Good *et al*., 2004).

Improved NUE in canola (Good *et al*., 2007) and rice (Shrawat *et al*., 2008) has been demonstrated by overexpression of a barley alanine aminotransferase (*HvAlaAT*, Muench and Good, 1994) driven by a *Brassica* turgor‐responsive promoter (p*Btg*) and a rice stress‐inducible promoter (p*OsAnt1*), respectively. In canola, transgenic events have been tested under field conditions in the United States and shown to impart NUE under limiting N conditions (Good *et al*., 2007). Shrawat *et al*. (2008) and Beatty *et al*. (2013) conducted studies to determine the effects of overexpression of *HvAlaAT* in transgenic rice (cv. Nipponbare). They reported increased biomass, grain yield and N content in plants that were grown under controlled glasshouse/growth chamber conditions. While these data are reflective of the data reported in the dicot Brassica, it was important to see whether any rice glasshouse phenotypes were reflected in the field, as expression and performance can vary significantly with the inherent biotic and abiotic pressures associated with field growth.

In this study, we report the production of marker‐free *HvAlaAT* transgenic lines in a New Rice for Africa (NERICA) background and present the results of confined field trials in two locations. Our results clearly showed that pOsAnt1:*HvAlaAT* overexpression significantly increased plant biomass and grain yield through an increased number of productive tillers compared to controls under limited N supply. These findings imply expression of *HvAlaAT* in rice can improve rice agronomic traits and economic characteristics and could be an efficient way to boost low‐input rice production systems. The work presented here discusses the application of this technology in the African rice variety NERICA4 with the eventual goal of supplying marker‐free NUE seeds to African farmers.

## Results

### Production of marker‐free transgenic plants

**Figure 1:**
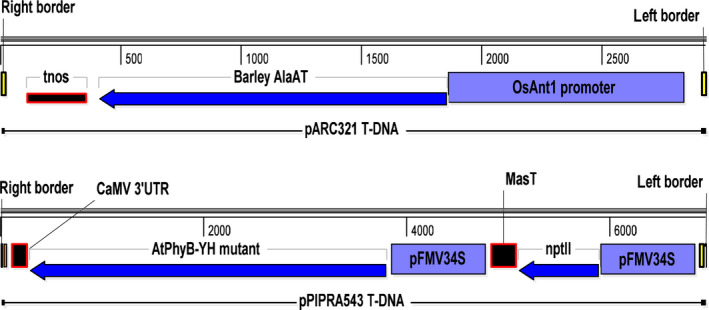
T‐DNA of binary vectors pARC321 and pPIPRA543 used for cotransformation of NERICA4. pOsAnt1, promoter of the rice antiquitin 1 gene; *HvAlaAT*, barley alanine aminotransferase gene; tnos, 3′UTR of nopaline synthase gene; pFMV34S, promoter of the figwort mosaic virus 34S gene; *nptII*, neomycin phosphotransferase gene; MasT, 3′UTR of mannopine synthase gene; *AtPhyB‐YH*, YH‐mutant of *Arabidopsis* phytochrome B gene; CaMV 3′UTR, 3′UTR of cauliflower mosaic virus 35S gene.

To obtain NUE NERICA4 rice plants for potential commercial applications, we produced plants expressing *HvAlaAT* from a stress‐inducible rice antiquitin promoter, p*OsAnt1*, by *Agrobacterium*‐mediated cotransformation using two binary vectors, pARC321 and pPIPRA543 (Figure 1). Plants were screened for single, simple insertions without the presence of any miscellaneous vector sequences and from which the selectable marker could be removed by segregation. Results from cotransformation of NERICA4 and molecular selection of events are summarized in Table S1.

**Figure 2:**
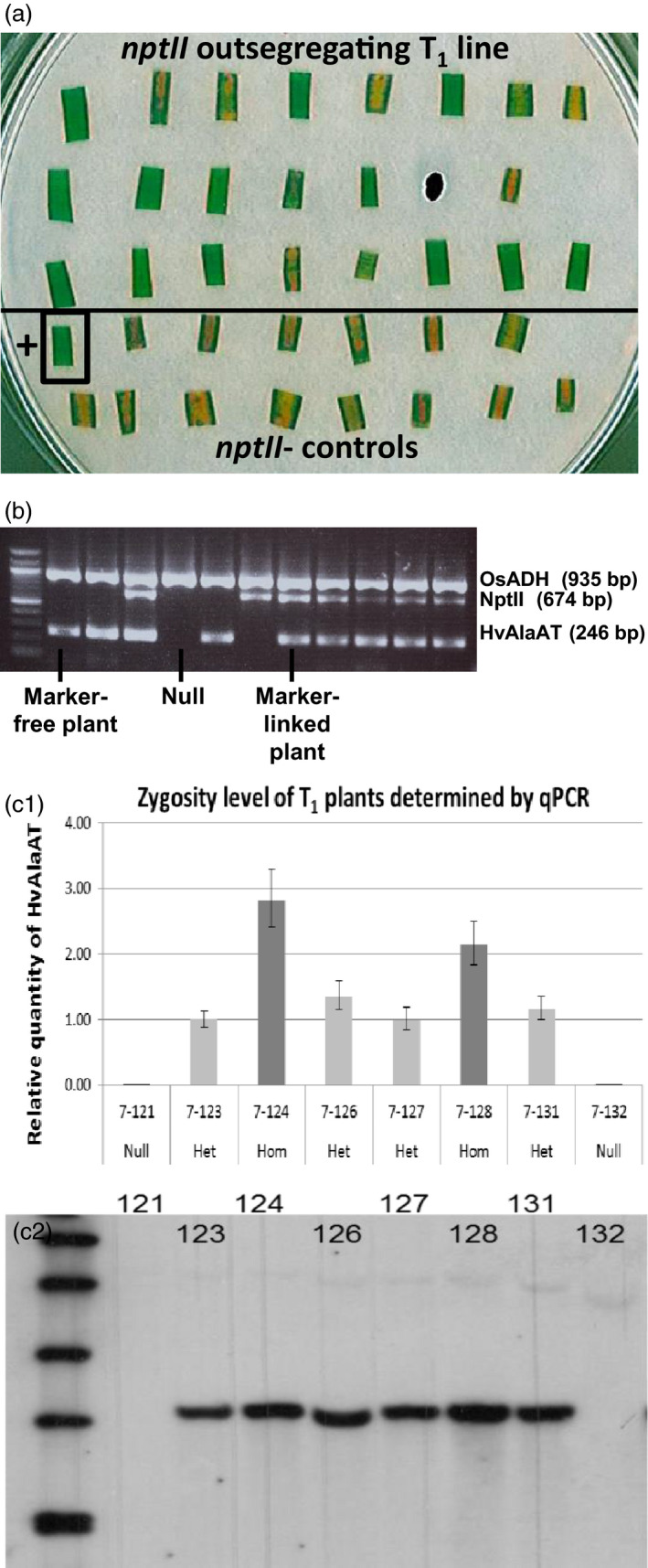
(a) Leaf disc test on G418‐containing media plate with a T_1_ line segregating for *nptII*. Six of 22 leaf discs above the line show yellowing similar to the *nptII*‐negative control discs in the bottom part; 16 leaf discs show no yellowing, similar to the *nptII*‐positive control disc (bottom left). Yellowing discs are traced back to their seedlings of origin, which were maintained as selectable marker‐free T_1_ plants. Subsequent PCR on leaf material confirmed the absence of the *nptII* gene. (b) Confirmation of marker segregation in event NUE‐1 by PCR using *HvAlaAT* and *nptII* gene‐specific primers. Six T1 plants are *HvAlaAT*‐positive/*nptII*‐positive, three plants are *HvAlaAT*‐positive/marker‐free, one plant is *HvAlaAT*‐negative/*nptII*‐positive, and one plant is a sibling null. (c) Analysis of *HvAlaAT* zygosity level in NUE‐1 T_1_ plants. c1: *HvAlaAT* copy number determined by relative quantification (RQ) using qPCR; plants with RQ >2 are considered homozygous (error bars are standard deviations from three replicates; Het = heterozygous, Hom = homozygous); c2: confirmation of single insertion of *HvAlaAT* by Southern blot analysis; genomic DNA of eight lines digested with *EcoRI* and probed with *HvAlaAT* fragment.

A mutated phytochrome B gene (*AtPhyB‐YH*) in pPIPRA543 was to be used as a visual marker for selection against the presence of the selectable marker T‐DNA. In *Arabidopsis* and japonica rice seedlings germinated in the dark, an easily scorable short‐hypocotyl (coleoptile) phenotype (Su and Lagarias, 2007; Chi‐Ham, PIPRA (Public Intellectual Property Resource for Agriculture), pers com) is observed from *AtPhyB‐YH* expression. However, NERICA4 rice did not display this phenotype; thus, a G418 leaf disc method (Figure 2a) was used to identify events with segregation and loss of pPIPRA543 T‐DNA. Fully characterized (Figure 2b, c) homozygous, single copy, marker‐free seed from six events was used for field trials.

### Overexpression of *HvAlaAT* increases grain yield under limited N conditions

The field performance of pOsAnt1:*HvAlaAT* NERICA4 transgenic lines was evaluated for two growing seasons in a paddy field (lowland conditions) under three different N treatments. Soil residual N was depleted prior to the trials to keep the background as low as possible; N was applied to the plots at 100% (N_100%_), 50% (N_50%_) and 0% (N_0%_; no application) of the local farm rate, 180 kg/ha N.

#### First paddy field experiment, CIAT, Palmira, Colombia

During the first growing season (August–December, 2012), homozygous T_3_ lines of six independent pOsAnt1:*HvAlaAT* events were evaluated with NERICA4 wild‐type (WT) and local varieties Curinga and Fedearroz174 as controls. The average soil residual N, measured before the trial, was relatively low, with NH_4_^+^ varying between 2.9 and 4.0 mg/kg and NO_3_^−^ between 0.2 and 0.4 mg/kg (Table S2). Climatic data are shown in Figure S1.

**Figure 3:**
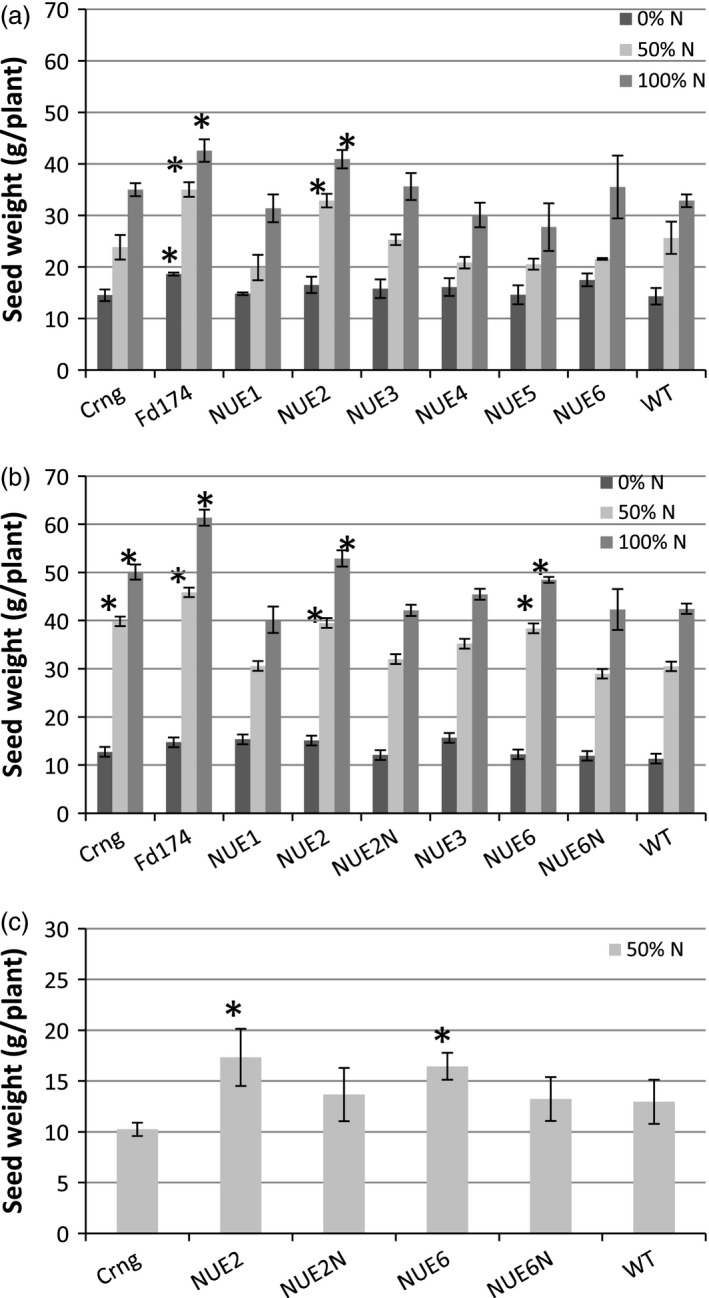
Average plant seed yields of pOsAnt1*‐HvAlaAT* events, null siblings and two local varieties compared to WT under different nitrogen fertilization regimes. (a) 2012 trial at CIAT, Palmira; (b) 2013 trial at CIAT, Palmira; (c) 2013‐14 rain‐fed trial at Santa Rosa. Values are means ±SD of 21 (a and b) or 18 plants. *indicates a significant yield difference from WT at *P* < 0.05.

Significant differences were detected in N treatment (N), genotype (G) and N × G interaction for most of agronomic traits studied (Table 1). Overall, the trial displayed a good linear response to N fertilization (Figure 3a) with WT grain yield increasing from 14.3 g/plant (N_0%_) to 32.8 g/plant (N_100%_). Yield of all genotypes also responded to N with event NUE‐2 significantly outperforming other events and WT at both N_50%_ and N_100%_, essentially performing similar to the high‐yielding local cultivar Fedearroz174 (Colombian Rice Farmers Federation).

**Table 1:** Yield parameters measured from 2012 paddy field trial at CIAT, Palmira. GY, grain yield (g/plant); GW, grain weight (g); PH, plant height (cm); TN, tiller number; PN, panicle number; DB, dry weight biomass (g/plant); PL, panicle length (cm). Values are means ± SD from 21 plants

2012								
Genotype	Treatment	GY	1000 GW	PH	TN	PN	DB	PL
WT	N 0%	14.30 ± 1.62	24.37 ± 0.42	96.05 ± 3.79	4.14 ± 0.38	4.10 ± 0.41	8.74 ± 1.52	22.12 ± 1.26
	N 50%	25.64 ± 3.12	25.50 ± 0.50	109.68 ± 1.87	7.19 ± 0.68	7.10 ± 0.59	23.27 ± 11.25	23.02 ± 1.39
	N 100%	32.84 ± 1.22	27.03 ± 0.55	117.35 ± 3.54	8.76 ± 0.16	8.19 ± 0.41	22.18 ± 1.55	25.76 ± 0.30
NUE‐1	N 0%	14.80 ± 0.27	25.53 ± 0.38^a^	78.77 ± 1.07^c^	6.00 ± 0.38^a^	5.95 ± 0.30^a^	10.07 ± 0.44	18.74 ± 0.27^c^
	N 50%	19.89 ± 2.47^c^	26.47 ± 0.58^a^	94.48 ± 3.16^c^	7.05 ± 0.70	6.95 ± 0.79	14.22 ± 1.18^c^	22.52 ± 0.68
	N 100%	31.36 ± 2.71	27.40 ± 0.26	109.48 ± 1.46^c^	10.71 ± 1.76^a^	10.33 ± 1.54^a^	20.81 ± 1.52	24.55 ± 0.62
NUE‐2	N 0%	16.51 ± 1.59	24.90 ± 0.10	84.81 ± 1.46^c^	6.10 ± 0.79^a^	6.00 ± 0.87^a^	10.20 ± 1.40	20.40 ± 0.81^c^
	N 50%	32.87 ± 1.31^a^	26.50 ± 0.36^a^	104.94 ± 1.83^c^	9.57 ± 0.38^a^	9.48 ± 0.44^a^	18.97 ± 0.83	22.79 ± 0.12
	N 100%	40.92 ± 1.78^a^	28.20 ± 0.20^a^	113.53 ± 2.34^c^	11.91 ± 0.23^a^	11.90 ± 0.46^a^	22.83 ± 1.44	24.19 ± 1.16^c^
NUE‐3	N 0%	15.79 ± 1.81	25.37 ± 0.21^a^	88.10 ± 2.16^c^	4.76 ± 0.44	4.67 ± 0.46	9.78 ± 1.53	21.86 ± 1.01
	N 50%	25.27 ± 1.04	26.27 ± 0.25^a^	99.37 ± 1.82^c^	7.76 ± 0.81	7.71 ± 0.76	17.36 ± 2.07	21.76 ± 0.68
	N 100%	35.60 ± 2.60	27.80 ± 0.72^a^	110.52 ± 2.79^c^	10.00 ± 0.89	9.81 ± 0.70^a^	21.62 ± 1.70	23.71 ± 0.25^c^
NUE‐4	N 0%	16.08 ± 1.71	26.43 ± 0.32^a^	90.54 ± 4.31^c^	5.29 ± 0.52	5.24 ± 0.50	12.35 ± 1.57	21.90 ± 1.90
	N 50%	20.83 ± 1.12^c^	27.50 ± 0.44^a^	96.44 ± 0.67^c^	6.62 ± 1.00	6.57 ± 1.00	15.78 ± 2.94	21.90 ± 0.66
	N 100%	30.06 ± 2.37	28.67 ± 0.65^a^	111.07 ± 1.84^c^	9.33 ± 0.58	9.10 ± 0.46	23.66 ± 1.40	24.32 ± 0.43^c^
NUE‐5	N 0%	14.59 ± 1.82	25.70 ± 0.30^a^	59.86 ± 2.34^c^	6.95 ± 1.00^a^	6.76 ± 0.79^a^	8.78 ± 1.26	21.33 ± 0.87
	N 50%	20.51 ± 1.07^c^	26.97 ± 0.06^a^	68.42 ± 4.11^c^	10.29 ± 0.43^a^	9.81 ± 0.59^a^	15.15 ± 1.03^c^	21.02 ± 0.55^c^
	N 100%	27.73 ± 4.62^c^	28.37 ± 0.32^a^	80.24 ± 2.04^c^	13.14 ± 1.22^a^	12.66 ± 1.41^a^	17.82 ± 1.72	22.77 ± 0.20^c^
NUE‐6	N 0%	17.47 ± 1.23	25.57 ± 0.32^a^	85.89 ± 1.50^c^	7.00 ± 1.41^a^	6.81 ± 1.21^a^	12.18 ± 0.39	22.87 ± 1.40
	N 50%	21.55 ± 0.18^c^	26.70 ± 0.26^a^	90.30 ± 3.35^c^	7.81 ± 0.58	7.67 ± 0.54	15.77 ± 0.50	23.26 ± 0.65
	N 100%	35.52 ± 6.08	28.17 ± 0.49^a^	106.41 ± 4.12^c^	11.67 ± 0.95^a^	11.24 ± 0.84^a^	23.03 ± 2.99	25.21 ± 0.31
Curinga	N 0%	14.51 ± 1.13	25.77 ± 0.15^a^	86.92 ± 2.38^c^	6.63 ± 0.22^a^	6.53 ± 0.09^a^	11.07 ± 1.00	20.41 ± 0.73^c^
	N 50%	23.81 ± 2.39	26.17 ± 0.38^a^	99.33 ± 3.10^c^	8.76 ± 0.36^a^	8.57 ± 0.14^a^	16.27 ± 2.15	22.83 ± 0.57
	N 100%	34.99 ± 1.26	26.97 ± 0.25	113.43 ± 0.62^c^	12.33 ± 1.31^a^	11.65 ± 0.88^a^	23.70 ± 1.30	24.26 ± 0.65^c^
Fedearroz 174	N 0%	18.62 ± 0.28^a^	25.20 ± 0.60^a^	76.48 ± 0.93^c^	10.05 ± 0.59^a^	9.90 ± 0.46^a^	14.56 ± 0.73^a^	23.29 ± 0.45
	N 50%	35.00 ± 1.42^a^	26.37 ± 0.21^a^	90.38 ± 1.81^c^	12.38 ± 0.30^a^	12.19 ± 0.22^a^	25.11 ± 3.91^a^	24.93 ± 0.89^a^
	N 100%	42.56 ± 2.17^a^	28.20 ± 0.62^a^	95.63 ± 0.91^c^	15.46 ± 0.97^a^	15.03 ± 1.24^a^	33.83 ± 2.64^a^	25.19 ± 1.09
N Level (N)		b	b	b	b	b	b	b
Genotype (G)		b	b	b	b	b	b	b
N × G		b	a	b	ns	ns	a	b
CV%		8.5	1.5	2.4	9.6	9.1	15.4	3.6

a Significantly higher than WT *P* < 0.05.

b Significantly higher than WT at *P* < 0.01.

ns = not significant *P* > 0.05.

c Significantly lower than WT at *P* < 0.05 LSD0.05 for GY = 3.53.

Other agronomic traits like plant height, tiller number, panicle number, panicle length, 1000 grain weight and biomass were clearly affected by the N treatments (Table 1). At N_0%_, all transgenic lines flowered earlier compared to other N treatments (data not shown). Under all N levels, 1000GW was increased in all transgenic events as compared to WT. Both tiller and panicle numbers were affected by N, decreasing with reduced N, and were highly correlated. Events NUE‐2 and NUE‐5 maintained higher tiller and panicle numbers as compared to WT irrespective of N levels; for NUE‐1 and NUE‐6, higher tiller and panicle numbers were observed at N_0%_ and N_100%_. NUE‐3 and NUE‐4 showed little difference in these traits as compared to WT. In NUE‐2, the increased tiller and panicle numbers appear to support a higher grain yield (at N_50%_ and N_100%_) as they do for the variety Fedearroz. Aboveground biomass decreased dramatically with lower N in all of the genotypes tested. Although some events trended towards increased biomass compared to WT (NUE‐4 and NUE‐6 at N_0%_), none of these differences were significant (Table 1). Panicle length was less affected by decreasing N levels; compared to WT, most transgenic events had shorter panicles. Under all N rates, transgenic events were shorter in plant height than WT, with NUE‐5 visually showing a dwarf phenotype (data not shown).

Chlorophyll content was determined at three different time points (flowering, milky and dough stage) during the crop cycle (Table S3). The N × G interaction was significant in milky and dough stage. It was observed at the later developmental dough stage that two events (NUE‐4 and ‐5) maintained higher chlorophyll content than WT, under limiting N. NUE‐6 stayed greener than WT from milky stage onwards, under all N treatments. It is interesting to note that this event had higher chlorophyll content than WT, specifically at N_0%_ for all stages, which could be a contributing factor to its higher grain yield compared to WT.

#### Second paddy field experiment, CIAT, Palmira, Colombia

During the second growing season (March–July, 2013), homozygous T_3_ lines of the four better performing independent events from 2012 (NUE‐1, ‐2, ‐3 and ‐6), together with two sibling nulls (NUE‐2N and ‐6N), WT and local controls, were evaluated. Similar to 2012, the N and G effects were significant at *P* < 0.01 (Table 2).

**Table 2:** Yield parameters measured from 2013 paddy field trial at CIAT, Palmira. Parameters are identical to Table 1. Values are means ±SD from 21 plants

2013								
Genotype	Treatment	GY	1000 GW	PH	TN	PN	DB	PL
WT	N 0%	11.33 ± 0.28	24.87 ± 0.29	95.29 ± 4.72	4.48 ± 0.08	4.33 ± 0.08	8.13 ± 0.38	21.48 ± 1.08
	N 50%	30.50 ± 1.17	26.56 ± 0.39	116.87 ± 2.07	9.21 ± 0.26	8.60 ± 0.05	26.93 ± 0.58	25.27 ± 0.62
	N 100%	42.44 ± 1.06	27.98 ± 0.04	133.29 ± 1.55	11.43 ± 0.76	11.14 ± 0.99	35.70 ± 0.47	26.92 ± 0.57
NUE‐1	N 0%	15.34 ± 4.03	25.27 ± 0.19	91.90 ± 9.28	5.86 ± 1.61	5.86 ± 1.61	12.65 ± 4.78^a^	19.92 ± 0.07^c^
	N 50%	30.58 ± 2.16	26.81 ± 0.17	113.10 ± 1.01	9.76 ± 0.92	9.71 ± 0.87	31.95 ± 0.54^a^	24.21 ± 0.57
	N 100%	40.16 ± 2.73	28.22 ± 0.02	119.38 ± 1.57^c^	11.81 ± 1.05	11.29 ± 0.76	38.91 ± 3.68	26.38 ± 0.72
NUE‐2	N 0%	15.09 ± 2.10	25.72 ± 0.05^a^	88.33 ± 4.13^c^	5.52 ± 0.36	5.52 ± 0.36	10.46 ± 0.61	20.59 ± 0.68
	N 50%	39.49 ± 3.61^a^	26.84 ± 0.10	113.52 ± 1.95	13.00 ± 1.25^a^	12.62 ± 1.53^a^	34.91 ± 3.50^a^	24.12 ± 0.55
	N 100%	52.90 ± 1.70^a^	28.48 ± 0.25	123.09 ± 2.93^c^	15.33 ± 0.54^a^	14.81 ± 0.64^a^	43.69 ± 0.95^a^	25.76 ± 0.69
NUE‐2N	N 0%	12.09 ± 0.38	24.98 ± 0.30	90.00 ± 4.11^c^	5.10 ± 0.79	4.43 ± 0.14	8.39 ± 1.90	20.31 ± 0.60
	N 50%	31.99 ± 4.70	26.48 ± 0.37	114.90 ± 4.58	9.43 ± 1.76	9.33 ± 1.59	26.77 ± 3.68	24.24 ± 0.80
	N 100%	42.11 ± 1.15	27.89 ± 0.31	122.05 ± 1.64^c^	11.71 ± 0.29	11.62 ± 0.22	30.64 ± 1.44^c^	25.29 ± 0.38^c^
NUE‐3	N 0%	15.65 ± 4.88	25.71 ± 0.05^a^	93.24 ± 3.39	5.52 ± 1.43	5.52 ± 1.43	11.93 ± 4.18	20.00 ± 0.74
	N 50%	35.21 ± 8.68	26.54 ± 0.88	115.10 ± 2.84	9.71 ± 1.36	9.62 ± 1.51	29.26 ± 6.54	23.83 ± 1.91
	N 100%	45.46 ± 1.15	28.12 ± 0.12	125.67 ± 0.92^c^	12.71 ± 0.25	12.52 ± 0.46	37.40 ± 2.02	25.40 ± 0.54
NUE‐6	N 0%	12.24 ± 2.98	25.11 ± 0.20	76.48 ± 4.65^c^	5.14 ± 1.08	5.03 ± 1.12	10.49 ± 1.79	20.44 ± 0.88
	N 50%	38.39 ± 2.22^a^	26.72 ± 0.18	111.03 ± 2.29^c^	11.62 ± 0.79^a^	11.57 ± 0.86^a^	35.02 ± 0.75^a^	25.95 ± 0.64
	N 100%	48.45 ± 0.61^a^	28.52 ± 0.21	115.76 ± 1.37	15.33 ± 0.43^a^	14.99 ± 0.38^a^	41.13 ± 0.86^a^	27.09 ± 0.45
NUE‐6N	N 0%	11.92 ± 0.31	25.14 ± 0.29	85.84 ± 3.07^c^	5.24 ± 0.58	4.43 ± 0.29	8.44 ± 0.55	21.10 ± 0.85
	N 50%	28.95 ± 2.89	26.53 ± 0.15	109.12 ± 1.31^c^	10.18 ± 1.09	9.81 ± 1.31	27.90 ± 2.59	26.27 ± 1.01
	N 100%	42.31 ± 4.26	27.87 ± 0.40	112.52 ± 0.95^c^	12.48 ± 1.89	12.38 ± 1.90	34.02 ± 6.55	27.52 ± 1.15
Curinga	N 0%	12.74 ± 0.59	25.14 ± 0.12	90.24 ± 0.58^c^	5.81 ± 0.46	5.76 ± 0.44	11.24 ± 1.11	21.76 ± 1.54
	N 50%	39.84 ± 0.22^a^	25.84 ± 1.32^c^	113.05 ± 2.07	13.26 ± 0.66^a^	13.48 ± 0.41^a^	39.48 ± 1.96^a^	24.50 ± 1.34
	N 100%	50.08 ± 1.58^a^	28.63 ± 0.06^a^	118.80 ± 2.22^c^	16.33 ± 0.58^a^	16.27 ± 0.64^a^	45.07 ± 2.07^a^	25.00 ± 1.32^c^
Fedearroz 174	N 0%	14.73 ± 3.35	25.67 ± 0.44^a^	80.24 ± 1.59^c^	9.10 ± 1.19^a^	8.95 ± 1.33^a^	13.88 ± 2.33^a^	22.65 ± 0.86
	N 50%	45.86 ± 1.39^a^	26.29 ± 0.17	102.19 ± 2.97^c^	15.62 ± 0.54^a^	15.12 ± 0.49^a^	42.53 ± 1.07^a^	25.22 ± 0.58
	N 100%	61.36 ± 1.67^a^	28.23 ± 0.15	112.20 ± 1.93^c^	19.81 ± 0.22^a^	18.83 ± 0.61^a^	53.74 ± 0.96^a^	25.89 ± 0.34
N Level (N)		b	b	b	b	b	b	b
Genotype (G)		b	a	b	b	b	b	b
N × G		b	ns	b	b	b	b	ns
CV%		9	1.4	2.4	8.9	9.4	9.6	3.9

a Significantly higher than WT *P* < 0.05.

b Significantly higher than WT at *P* < 0.01.

ns = not significant *P* > 0.05.

c Significantly lower than WT at *P* < 0.05 LSD0.05 for GY = 4.72.

Although the same N treatments and conditions were repeated in the second season, the overall agronomic performance of the 2013 trial was consistently better than the 2012 trial in terms of grain yields and tiller and panicle numbers. However, the trend of genotypic response to N treatments was quite similar (Table 2).

**Figure 4:**
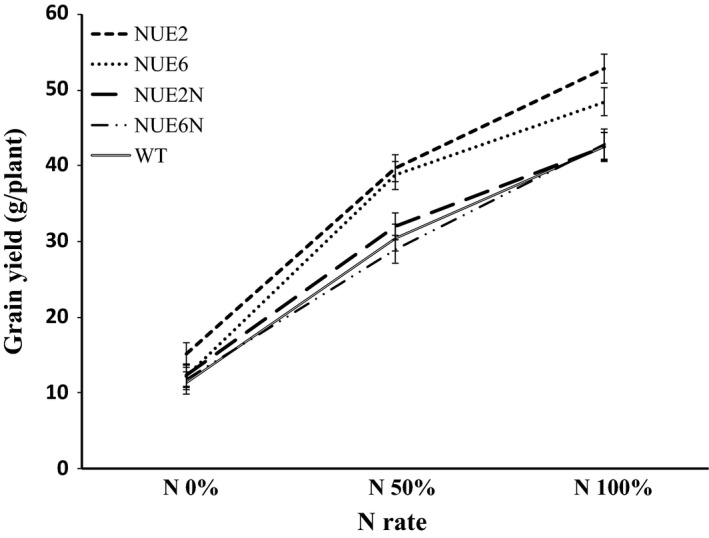
Response of NUE‐2 and NUE‐6 and their sibling nulls for grain yield at different N application rates during the 2013 trial. Values are means ±SD for 21 plants.

Grain yield increased for all genotypes with increasing N, and there were no differences in yield between WT and the two sibling nulls, NUE‐2N and NUE‐6N, under any N rate (Figure 3b). Lead event NUE‐2 showed an increase in grain yield by 29.5% and 24.6%, respectively, at N_50%_, and N_100%_, as compared to WT. Similarly, the other lead event, NUE‐6, outyielded WT by 25.9% and 14.2%, respectively, at N_50%_, and N_100%_. The N‐to‐yield response curves of NUE‐2 and NUE‐6 compared to their sibling nulls are shown in Figure 4. NUE‐6 had higher grain yield at N_50%_ and NUE‐2 at all three N treatments, with the largest percentage change apparent at N_50%_.

Unlike in 2012, the 1000GW of the events did not generally differ from WT or sibling nulls (Table 2), whereas plant height continued to be impacted in the transgenic lines and the nulls. When compared to their sibling nulls, however, NUE‐2 and NUE‐6 did not significantly differ in height, except for NUE‐6 at N_0%_ where the null was taller. Compared to WT or their respective nulls, tiller and panicle number of NUE‐2 and NUE‐6 were increased at N_50%_ and N_100%_ (Table 2). Panicle length showed response to N levels across all three treatments, but in contrast to 2012, the trend of shorter panicles in events was much reduced.

Along with grain yield increase, NUE‐2 and NUE‐6 showed an increase in biomass at N_50%_ and N_100%_ compared to WT, representing a 29.6%–22.4% and 30.0%–15.2% increase, respectively. NUE‐1 showed a significant biomass increase compared to WT, but the increase is not coupled to an increase in grain yield (Table 2). Chlorophyll content was higher in 2013 at the earlier milky developmental stage for NUE‐2 at all N rates and NUE‐3 and NUE‐6 at limiting N (Table S3).

Nitrogen concentration at flowering stage in leaves of NUE‐2 and NUE‐6 was significantly increased at all N treatments (Table 3) compared to WT and to a lesser extent at the later dough stage. N concentration in grain was only measured for NUE‐2, its sibling null and WT. At limiting N rates, the N concentration was not significantly higher in NUE‐2 grains.

**Table 3:** Leaf N (at flowering and dough stage) and grain N concentration (at mature stage) in lead events under different N regimes in the 2013 trial, CIAT, Palmira. Values (mg/g) are means ±SD of three plants

2013				
Genotype	Treatment	Leaf N @Flwr	Leaf N @Dough	Grain N
WT	N 0%	13.69 ± 1.04	8.83 ± 0.50	11.23 ± 0.98
	N 50%	18.96 ± 2.02	11.05 ± 1.18	11.90 ± 0.26
	N 100%	24.14 ± 1.87	12.26 ± 0.56	12.36 ± 0.21
NUE‐2	N 0%	15.86 ± 0.46^a^	10.15 ± 1.66	12.25 ± 1.07
	N 50%	21.15 ± 1.69^a^	13.77 ± 1.19^a^	12.70 ± 0.26
	N 100%	28.36 ± 2.63^a^	16.89 ± 1.53^a^	14.01 ± 0.62^a^
NUE‐2N	N 0%	15.41 ± 0.61	6.95 ± 1.35	11.56 ± 1.57
	N 50%	20.99 ± 0.54	10.31 ± 0.88	11.85 ± 0.11
	N 100%	25.00 ± 0.30	12.89 ± 0.36	12.73 ± 0.27
NUE‐6	N 0%	20.07 ± 0.70^a^	8.88 ± 2.44	No data
	N 50%	21.93 ± 1.54^a^	12.29 ± 2.87	
	N 100%	29.18 ± 2.50^a^	15.78 ± 1.52^a^	
NUE‐6N	N 0%	15.06 ± 1.18	7.65 ± 0.68	No data
	N 50%	20.32 ± 2.05	10.53 ± 0.75	
	N 100%	24.94 ± 1.58	12.00 ± 0.86	
N Level (N)		b	b	a
Genotype (G)		b	b	a
N × G		ns	ns	ns
CV%		5.8	12	6.8

a Significantly higher than WT *P* < 0.05.

ns = not significant *P* > 0.05.

b Significantly higher than WT at *P* < 0.01.

#### Rain‐fed upland field experiment, Santa Rosa, Colombia

A third trial was conducted to test the performance of the two lead events, NUE‐2 and NUE‐6, this time under rain‐fed upland conditions. T_4_ seed along with null and WT seed was direct‐sown on dry soil supplemented with only one N rate (N_50%_) and grown to maturity (November 2013–March 2014). Besides limited N, the plants were also exposed to natural (moderate) drought stress flanking the heading stage. Soil moisture level decreased from 60% to 25% in the top 20 cm during grain filling (Figure S2). In addition, the soil physical and chemical characteristics at this upland trial site were different from the lowland site: higher in organic matter, residual N (NH_4_^+^ and NO_3_^−^) and % clay; lower in P, K and pH (Table S2).

Table 4 shows there was an overall 50% yield reduction for WT (13 g/plant) compared to the paddy field experiments (28 g/plant), which may be attributed to limited water supplementation during the crop season. The sibling nulls and WT did not differ from each other in grain yield, whereas yield of the local variety Curinga was 20% lower than WT (Figure 3c). Despite the conditions, the maintenance of yield of the lead events under limiting N was comparable to lowland growing conditions NUE‐2 and NUE‐6 displayed significantly higher grain yield at N_50%_; 33.8% and 26.9%, respectively, as compared to WT, and 26.8% and 24.4% over their respective sibling nulls (Table 4; Figure 3c).

**Table 4:** Yield parameters measured from 2013 to 2014 rain‐fed field trial at Santa Rosa; DF50%, number of days to 50% flowering; Y, estimated yield from plot yield (kg/ha); other yield parameters are identical to Table 1. Values are means ±SD of 18 plants

	Santa Rosa rain‐fed 2013–2014					
Genotype	GY	DF50%	PH	PN	PL	Y
NUE‐2	17.33 ± 2.81^a^	67.67 ± 1.16^a^	90.22 ± 6.43	6.56 ± 0.78^a^	23.63 ± 17.33	3678.10 ± 93.62^a^
NUE‐2N	13.67 ± 2.62	66.33 ± 0.57	91.00 ± 3.22	5.50 ± 0.87	23.42 ± 13.67	2590.92 ± 16.63
NUE‐6	16.44 ± 1.33^a^	66.67 ± 0.57	83.89 ± 1.84^b^	6.33 ± 0.29^a^	23.61 ± 16.44	3682.52 ± 77.75^a^
NUE‐6N	13.22 ± 2.15	66.67 ± 0.57	80.06 ± 2.34^b^	5.72 ± 0.81	23.54 ± 13.22	2702.22 ± 118.58
WT	12.95 ± 2.16	66.11 ± 0.93	91.79 ± 3.24	5.10 ± 0.36	23.45 ± 12.95	2583.95 ± 163.14
Genotype	a	a	a	a	ns	a
CV%	12.3	1.2	4.6	9.8	4.0	18.9

a Significantly higher than WT *P* < 0.05.

b Significantly lower than WT at *P* < 0.05.

ns = not significant *P* > 0.05.

The yield advantage shown by the two NUE events was mainly due to the significant increase in panicle number (Table 4). As in the previous trial, transgenic and sibling null plant height tended to be shorter than WT.

### Hydroponic root experiment

**Figure 5:**
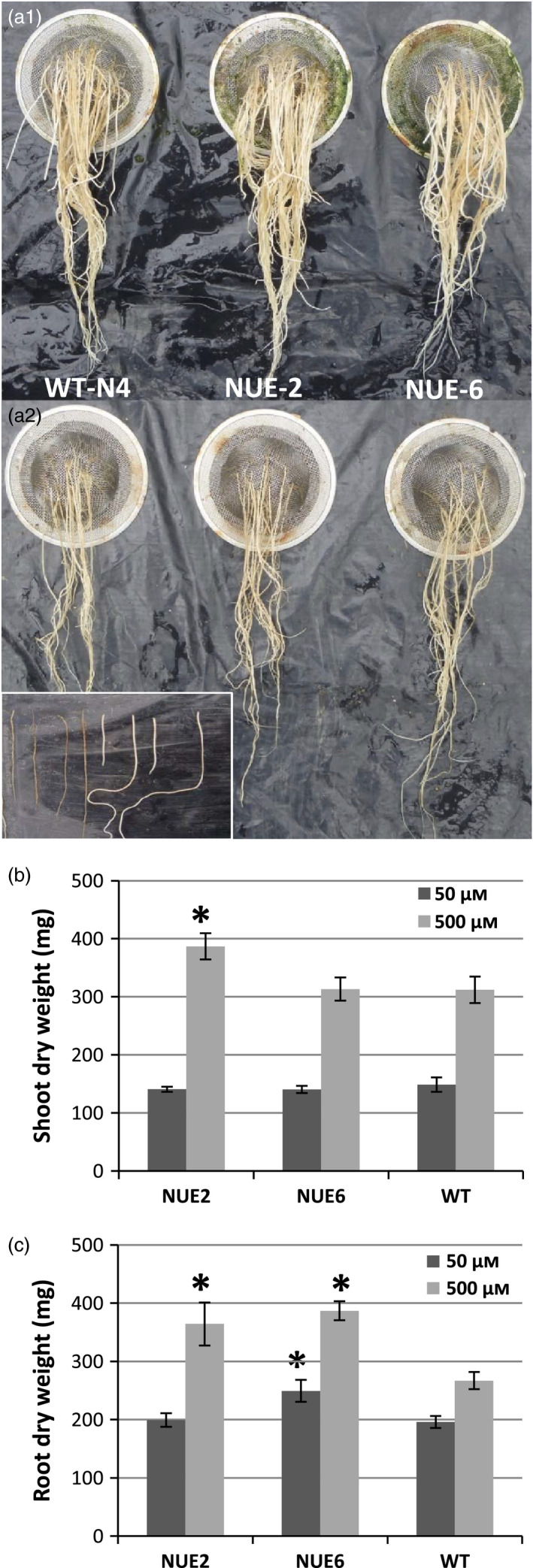
Comparison of root morphology (a), average shoot dry weight (b) and root dry weight (c) of two *pOsAnt1/HvAlaAT* events and WT NERICA4 rice plants growing in hydroponic solution containing ammonium as N source at different concentrations (a1 = 500 μm NH_4_^+^; a2 = 50 μm NH_4_^+^). Inset in a2 defines thin (left) and thick (right) roots as measured in Table 5. Values are means ± SD for 4 plants. *in b and c indicates a significant yield difference from WT at *P* < 0.05.

To investigate possible yield components or mechanisms, seedlings of lead events NUE‐2 and NUE‐6 were tested for altered plant growth phenotype in a hydroponic system with two different N concentrations in the form of NH_4_^+^, 50 and 500 μm, as described in previous hydroponic tests (Obara *et al*., 2011; Ogawa *et al*., 2014a,b). Significant differences between WT and transgenic lines were observed in 43‐day‐old plants (Table 5). NUE‐6 produced more branched roots than WT in both NH_4_^+^ concentrations (Figure 5a) and more root dry weight as a consequence (Table 5; Figure 5c). At 500 μm NH_4_^+^, NUE‐2 was taller and had an increased tiller number, more shoot and root biomass, and higher thick/thin root ratio than WT (Table 5; Figure 5). To obtain evidence for increased N uptake in transgenic lines, the total N content in dried shoot and root biomass was measured. By comparing the total N content from transgenic plants with WT, the increased N uptake efficiency at the 500 μm NH_4_^+^ rate for NUE‐2 and NUE‐6 was 54.2% and 77.6%, respectively. At the 50 μm rate, the increase in N uptake efficiency was less pronounced (Table 5).

**Table 5:** Yield parameters measured from hydroponic growth of two *OsAnt1‐HvAlaAT* lead events and WT NERICA4 under two NH_4_^+^ fertilization regimes. Values are means ±SD of four 43‐day‐old plants

	50 μm NH_4_^+^			500 μm NH_4_^+^		
	WT	NUE‐2	NUE‐6	WT	NUE‐2	NUE‐6
Plant height (mm)	367.5 ± 0.9	367.5 ± 0.5	357.5 ± 0.5	580.0 ± 2.5	597.5 ± 2.8	555.0 ± 3.4
Tiller number	1 ± 0	1 ± 0	1 ± 0	4 ± 0	4.5 ± 0.5	3.75 ± 0.5
Shoot dry weight (mg)	267.5 ± 31.6	260.75 ± 35.4	250.5 ± 25.4	1931.0 ± 181.1	2434.3 ± 366.8^a^	2127.5 ± 160.1
Root dry weight (mg)	287.3 ± 30.3	358.2 ± 122.5	400.0 ± 24.8^a^	1154.2 ± 115.0	1646.5 ± 218.1^a^	1712.0 ± 96.3^a^
Total root length (mm)	248.7 ± 45.5	250.03 ± 47.0	278.88 ± 45.5	343.13 ± 77.9	342.33 ± 35.0	364.80 ± 70.1
Shoot–root ratio	0.93 ± 0.08	0.80 ± 0.30	0.63 ± 0.10^b^	1.70 ± 0.31	1.35 ± 0.24	1.25 ± 0.15^b^
Thick–thin root ratio (%)	0.62 ± 0.09	0.72 ± 0.03	0.80 ± 0.04^a^	0.58 ± 0.01	0.78 ± 0.05^a^	0.81 ± 0.04^a^
Shoot N concentration (mg/g DW)	5.58 ± 1.23	6.75 ± 0.61	6.89 ± 0.32	12.62 ± 0.47	12.80 ± 2.83	18.94 ± 1.10^a^
Root N concentration (mg/g DW)	6.50 ± 0.77	7.43 ± 0.35	5.95 ± 1.43	5.23 ± 0.29	7.76 ± 0.70	7.89 ± 0.23
Total N shoot (mg)	1.50 ± 0.42	1.76 ± 0.28	1.73 ± 0.26	24.32 ± 1.68	34.07 ± 4.7^a^	40.41 ± 5.36^a^
Total N root (mg)	1.87 ± 0.31	2.65 ± 0.85	2.08 ± 0.52	6.04 ± 0.69	12.74 ± 1.71^a^	13.51 ± 1.04^a^
Total N content (mg)	3.37 ± 0.71	4.41 ± 0.86	3.81 ± 0.64	30.36 ± 1.17	46.82 ± 5.97^a^	53.93 ± 4.65^a^
Increase in N uptake efficiency (%)^c^		30.7	13.1		54.21	77.6

a Significantly higher than WT at *P* < 0.01.

b Significantly lower than WT at *P* < 0.01; both with Bonferroni correction.

c Increase in nitrogen uptake efficiency (%) = (total N transgenic − total N WT)/total N WT (Good *et al*., 2004).

### Gene transcription levels; metabolite and amino acid analysis and quantification

**Figure 6:**
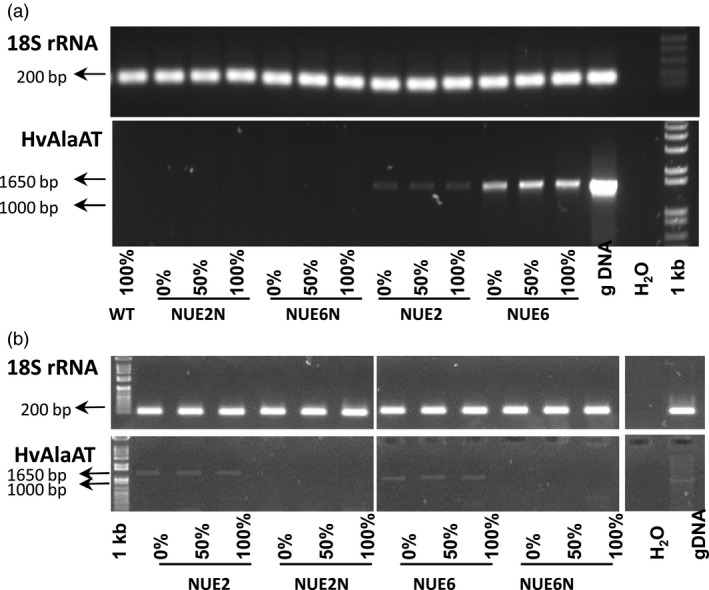
Gene expression analysis of pOsAnt1:*HvAlaAT* transgenic lines and controls evaluated under different N treatments in the 2013 trial. RT‐PCR analyses were performed using gene‐specific primers and RNA from leaf (a) and root (b) tissue at the flowering stage of development. The expression level of 18S rRNA was used as an internal control, and gDNA from the NUE‐2 transgenic event was used as a PCR control. To match the format of Figure b with a, Figure b is shown as a composite image of several rows of lanes that were run on one electrophoresis gel.

Field samples from rice grown during the 2nd paddy field trial (2013) were examined for gene function and metabolite changes in leaves and roots at the flowering stage. Endpoint PCR showed the presence of the *HvAlaAT* transcript in flag leaves with apparent higher levels of leaf expression in NUE‐6 than NUE‐2, although not quantitative (Figure 6a). Roots also showed evidence of RNA expression, although less pronounced (Figure 6b).

Leaf and root samples from the same 2013 trial were analysed for selected metabolites and amino acids. There were essentially no differences in leaf metabolites between the two lead transgenic events and WT at any N concentration, whereas the amino acids, glycine and valine, were significantly increased at the lower N application rates and alanine trended higher (Table S4).

Roots showed more differences overall, especially at lower N (Table S5). Metabolites shikimate, glycerate, malate, ribose and fructose were increased in one or both events at several N rates, while GABA, phosphoric acid and arabinose were only significantly increased in NUE‐2 at the lowest N. Fumarate was the only metabolite trending lower in roots of transgenic plants. Amino acids Glx (Glu+Gln), Asn, Thr, Gly, Ala and Val all significantly increased at low N in both events with Pro, Asp, Ile, Ser and Leu increasing only in NUE‐2.

To corroborate the amino acid data from field samples, we also sampled flag leaf and root tissue from the hydroponic experiment (Table S6). In transgenic leaves, higher levels of Gly and Ala were detected only at the high 500 μm NH_4_^+^ rate. In the roots at the low 50 μm NH_4_^+^ level, the same amino acids, Gly and Ala, were increased in NUE‐2, with only Ala increased in NUE‐6.

## Discussion

Marker‐free transgenic NERICA rice plants expressing *HvAlaAT* driven by a stress‐inducible promoter were produced and evaluated for yield increases under limiting N conditions in three field trials performed in Colombia. Our results presented here show yield increases and higher nitrogen use efficiency in this rice germplasm suitable for cultivation in Africa. Transgenic events showed up to a 30% yield increase compared to sibling nulls or WT when grown in paddy fields under limiting N_50%_ and a slightly lower, but still, significant yield increase (25%–30%) over their sibling nulls or WT when grown in rain‐fed conditions.

### Yield parameters are affected

The N, G and N × G interactions in the NERICA4 transgenic plants indicated significant differences for all agronomic traits studied (Tables 1, 2 and 3). Although N treatments and paddy field conditions were repeated for two seasons, the overall agronomic performance of the second trial (2013) was consistently better than the first trial (2012), with biomass and grain yield per plant being significantly higher in 2013. The observed variation is likely due to a number of environmental factors, but notably the concentration of soil available N prior to the experiment was different between the two trials (Table S2), likely due to ongoing mineralization and increased precipitation. Higher radiation was also observed in the second field trial (Figure S1), which via photosynthesis can affect total biomass, yield component traits and thus grain yield in rice (Singh, 2001). Regardless of these differences, the trend of genotype response to N treatment was very similar.

Overall, NUE‐2 and to a lesser extent NUE‐6 performed well in terms of grain yield. In addition to the increased grain yield, we observed a consistent increase in both tiller and panicle numbers in these events. These components are well known to be important determinants of grain yield in rice. The increased tiller number drove biomass values, which were higher for the lead events in 2013 as compared to WT, but not in 2012. Early biomass accumulation is consistent with the results obtained in canola field trials with plants overexpressing *HvAlaAT* (Good *et al*., 2007) where dry biomass increased as compared to WT under low N treatment and led to higher grain yields. Biomass accumulation was also noted in transgenic pOsAnt1‐*HvAlaAT japonica*‐type rice, with a 30%–35% increase as compared to controls (Shrawat *et al*., 2008). The accumulation of biomass however cannot solely be responsible for the subsequent increased grain yield, as NUE‐1 showed biomass accumulation in the lower N treatments in 2013, with no significant effect on grain yield (Table 2).

Other measured parameters and yield components—1000GW, panicle length and chlorophyll content—were not consistently different from the controls. In 2012, for example, all genotypes tested produced shorter panicles with large seeds. However, this phenotype was not observed in 2013 and 2014. NUE‐6 displayed late greenness in 2012, but less so in 2013. Plant height was consistently shorter for both transgenic and null siblings in the first two trials, but by the 3rd trial, NUE‐2 was displaying similar height as WT. Although short plant height is not consistent with the random and transient phenotype changes associated with somaclonal variation, we suspected this phenotype could still be caused by lingering tissue culture‐induced effects. The shorter plant height is not linked to the transgene as both transgenic and null plants displayed the phenotype. Alternatively, the NERICA varieties have been selected relatively recently and could possibly be segregating for some factors that control height. Observations of subsequent generations being trialled in sub‐Saharan Africa or backcrossing programme will shed more light on the persistence of the phenomenon.

### N‐to‐yield response and agronomic NUE

The N‐to‐yield response curves of the two lead events NUE‐2 and NUE‐6 (Figure 4) are consistent with the responses seen in the dicotyledonous canola where increases in yield are apparent under 50% less applied N (Good *et al*., 2007), with no difference in yield at very high or very low N. Evaluation of these types of response curves allows prediction of the optimal balance between lower N application and yield benefit under practical field crop situations. One question that needs to be answered is how such values can predict the NUE of the field crop. N available to the plant is difficult to measure, and many researchers have adjusted ‘applied fertilizer N’ when calculating NUE because not all fertilizer N is available to the plant, and applied N is not the only source of available N. Therefore, commonly used definitions do not provide a complete picture of NUE (Dawson *et al*., 2008). In the present study, the agronomic NUE (ANUE) parameter has been calculated based on the formula provided by Craswell and Godwin (1984). This parameter has proven to be selective for efficient NUE genotypes (Anbessa and Juskiw, 2012; Habtegebrial *et al*., 2013; Sui *et al*., 2013). Using this method, observed yields of NUE lead events were translated into highest ANUE under limited N treatment (N_50%_) (Table S7), which is consistent with the effect of the NUE gene.

### N content in biomass and seed

The conditions of our hydroponic studies are notably different from Shrawat *et al*. (2008) and Beatty *et al*. (2013), primarily with the much lower NH_4_^+^ concentrations used (50 and 500 μm vs. 4 mm, or 0.5, 2 and 5 mm, respectively) and the different growth stages of the plants during sampling. While our plants are quite small under the most limited N conditions (50 μm) and have a relatively low N concentration between 5.9 and 7.1 mg/g DW in roots, more biomass accumulates in the roots of pOsAnt1‐*HvAlaAT* plants vs. WT (Table 5). Under increased N (500 μm), more biomass and N are evident in both transgenic roots and shoots, similar in trend if not absolute values, to rice results reported by Shrawat *et al*., especially in shoots. The shoot/root ratio increases with higher N in the NERICA rice, but is lower in transgenic vs. WT plants in both N conditions, due to more root branching in transgenics. These events when grown in soil in the 2013 field trial showed significantly increased N content in leaves of flowering plants, even under limiting N application levels (Table 3). The measured increase in N content in grains of NUE‐2, however, was modest, and only at N_100%_. The combined hydroponic and field data suggest an early, efficient uptake and storage of N, leading to increased N in older leaves. Remobilization provides N for grain filling, resulting in more grain (increased yield), but not higher N concentration per grain, a response similar to transgenic canola where early biomass is increased, leading to increased grain yield, but identical grain composition (Good *et al*., 2007; Lu, unpublished).

### Metabolic changes

Similar to Beatty *et al*. (2013), expression of pOsAnt1‐*HvAlaAT* affected N leaf and root content in hydroponic growth (Table 5), but did not generally alter the content of amino acids in either transgenic event (Table S6), with the exception of glycine and alanine. The amino acids show significant increases in root (at 50 μm NH_4_^+^) and leaves (at 500 μm NH_4_^+^). The alanine accumulation could be a reflection of biosynthesis from the transgenic *HvAlaAT* or accumulation from endogenous alanine aminotransferases under potential hypoxic stress (Miyashita *et al*., 2007), which could be encountered in the hydroponic system. Substantially, more changes in amino acids and other metabolites were measured in the field‐derived root tissue of pOsAnt1‐*HvAlaAT* plants especially at the lowest N (Table S5). Significant accumulation of these compounds may be due to the higher stress of growing in N‐limited soil, and other abiotic stress parameters encountered in the field and give the plants an advantage in N accumulation, transport or remobilization. Despite the fact that this study and the previously published reports all examine effects of expression of *HvAlaAT* in rice, it is difficult to draw hard conclusions across the data primarily due to the different systems used for plant growth, including plant age, N concentrations, tissue collection and analysis. In our studies, the alterations in sugars (fructose and ribose) as well as amino acids, glutamine plus glutamate, and metabolism intermediates (shikimate and malate) seen in the field might suggest an alteration in the C/N balance that affects signalling consistent with suggestions by Beatty *et al*. (2013). Changes in glutamine plus glutamate content in field roots could be affecting initiation of lateral roots (Forde, 2014). Alternatively, other mechanisms such as altered photorespiratory cycling of N and C could be in play in the leaves (Peterhansel *et al*., 2010) and affect biomass accumulation. More directed and extensive analyses would be required to distinguish these mechanisms.

In summary, we present here the results of extensive confined field testing of transgenic rice overexpressing *HvAlaAT* and the responses of these plants to different levels of supplied N. Importantly, we evaluated the agronomic traits of these transgenic lines at all stages of plant growth in the field as a function of the environment. Our current findings suggest that early establishment of bushier and thickened roots is most likely primarily responsible for increased N scavenging in early plant stages. The resulting early storage of N in pOsAnt1‐*HvAlaAT* plants seems to provide the source for increased tiller and panicle number, leading to increased grain yield under limited N conditions. Transgenic events were identified that had significantly higher grain yield than sibling nulls and wild‐type controls under different N rates. The trait was stable over several rice growing ecologies in different years. The increased yield is furthermore correlated with altered metabolites and accumulation of N in late vegetative stages, but not in seed. Growing of this NUE crop should have significant potential economic and environmental benefits in both low‐ and high‐input agricultural systems. Using cotransformation, we were able to generate events in which the gene of interest T‐DNA segregated away from the SM T‐DNA, giving these transgenic plants an advantage in the path to commercialization. The marker‐free events selected in this study in Colombia are currently also being evaluated under confined field trial conditions in Ghana, Uganda and Nigeria, areas for which NERICA4 was selected.

## Experimental procedures

### Binary vectors and rice transformation

Two binary vectors were constructed for *Agrobacterium*‐mediated cotransformation (Figure 1). Vector pARC321 is a pPZP100‐derived binary vector (Hajdukiewicz *et al*., 1994), containing the 986‐bp OsAnt1 promoter (AK120185), the 1449‐bp cDNA of *HvAlaAT* (AK252381) and a 252‐bp 3′nos fragment.

The pPZP100‐derived binary vector pPIPRA543, containing a selectable marker and a visual selectable marker gene, was kindly donated by PIPRA (Chi‐Ham *et al*., 2012). One cassette contained *nptII*, controlled by the FMV34S promoter (Sanger *et al*., 1990) and 3′mas terminator. The other cassette contained a mutated phytochrome B sequence from *Arabidopsis thaliana* (*AtPhyB‐YH*; Su and Lagarias, 2007), controlled by pFMV34S and 3′CaMV.

pARC321 and pPIPRA543 were independently transformed into *Agrobacterium tumefaciens* strain EHA105 (Hood *et al*., 1993). For cocultivation, a layer of each culture was separately resuspended in liquid R2 medium (Ohira *et al*., 1973) and diluted to OD_600_ of 1.0. A 3 : 1 mix (pARC321: pPIPRA543) of cultures, with a final combined OD_600_ of approximately 1.0 was used for cocultivations. Transformation of rice variety NERICA4 (Africa Rice Center (WARDA)/FAO/SAA, 2008) using callus induced from mature dry seed was performed as described by Hiei *et al*. (1994). Healthy rooted T_0_ plantlets were potted into synthetic soil (Profile Greens Grade), acclimated for 1 week in growth chambers and grown to maturity in a glasshouse.

### Molecular characterization

Genomic DNA was extracted from 50 mg of young leaf tissue using a spin column extraction method, modified from the DNeasy 96 Plant Kit (Qiagen). Primer pairs used for PCR detection are shown in Table S8 for transgenes *HvAlaAT, nptII* and the control gene *OsADH*. Reactions were performed according to standard protocols using an annealing temperature of 60 °C.

A simplified leaf disc assay was developed to detect T_1_ seedlings that no longer contained the pPIPRA543 selectable marker due to insertion at separate loci and out segregation. Leaf segments of 7‐day‐old seedlings were cultured for 7 days on ½ strength MS plates, containing 100 mg/l G418 under a piece of sterile filter paper. G418‐sensitive, yellow leaf segments were detected 1 week later (Figure 2a) and confirmed to be *nptII* negative by PCR. Lines with ¼ of total seedlings being *nptII‐*negative were considered independent, single‐locus insertions and maintained for further growth in soil.

Copy number of T‐DNA insertions in T_0_ transgenic plants was estimated by real‐time quantitative PCR (qPCR) using primer pairs for *HvAlaAT* and rice *ubiquitin5* as reference gene (Table S8). For copy number estimation of the selective marker, an *nptII* primer pair was used (Weng *et al*., 2004) with rice *actin* (Jain *et al*., 2006) for normalization (Table S8). This qPCR protocol (annealing temperature of 58 °C) was also used to identify homozygous T_1_ plants, carrying a single copy insert.

Copy number of the transgenes was confirmed on selected plants by Southern blot hybridization, using HvAlaAT or nptII probes labelled with digoxigenin and DIG Luminescent Detection Kit (Roche).

RNA for RT‐PCR analysis was extracted from flag leaves and roots at flowering stage, from the 2013 field trial, using TRIzol reagent (Invitrogen‎) and reverse‐transcribed using DNase (Promega) and superscriptIII (Invitrogen). Endpoint PCR was conducted with primers listed in Table S8, using standard protocols and an annealing temperature of 55 °C. PCR products were checked on 1% agarose gel with SYBR‐safe stain.

### Hydroponic system and root architecture

Root phenotype was evaluated in hydroponic culture using a modified root basket method (Oyanagi *et al*., 1993) with two concentrations of NH_4_^+^ (50 and 500 μm; Obara *et al*., 2011; Ogawa *et al*., 2014a,b). Pregerminated seeds (30 °C, 2 days) were placed in baskets filled with river sand in boxes of water in a randomized block design with three replications. Seven days after sowing, the water was replaced with CIAT basal hydroponic solution of different NH_4_^+^ concentration at pH 6.5 (modified Subbarao *et al*., 2006). Plants were maintained in a glasshouse (average air temperature, 30 °C; average relative humidity, 50%; natural lighting). The nutrient solution was maintained at pH 5.5–6.5 and renewed every 3 days. At 43 days, dry root and shoot biomass, shoot height and N content of roots and shoots were measured.

### Design of confined paddy field experiment (2012 and 2013)

Paddy field experiments were performed at the confined field facility at CIAT, Palmira in Colombia (3°30′N, 76°21′W; 1000 mm annual rainfall, 965 m above sea level and annual average temperature 26 °C) in the dry season, August to December, 2012, and the rainy season, March to July, 2013. To deplete N from the trial sites, maize was cultivated over two prior consecutive seasons with no added fertilizer.

As quality control checks, we included two local varieties: Fedearroz174, a high‐yielding *indica*‐type lowland variety, and Curinga, an upland rain‐fed tropical *japonica*‐type variety.

The trial was planted in a split plot design with three replicates. Plots were 1.4 m^2^ with 20 cm between hills and 25 cm between rows, 28 plants per plot. Seedlings were transplanted from soil trays, one per hill, at 21 days after sowing (four‐leaf stage). The applied nitrogen treatments assigned into main plots included: (i) native (N_0%_), 0 kg/ha; (ii) 50% of farmer's practice (N_50%_) in Colombia (Berrio *et al*., 2002), 90 kg/ha, applied in three equal splits at the same time as N_100%_ treatment; (iii) farmer's practice (N_100%_) 180 kg/ha, applied in three equal splits: 60 kg/ha N as basal at 2 days after transplanting (DAT), 60 kg/ha N at 10 DAT and 60 kg/ha N at 30 DAT. To avoid N loss, every N split application was carried out over dry soil followed by a standing water layer. Other nutrients (70 kg KH_2_PO_4_, 60 kg KCl, 25 kg ZnSO_4_, 80 kg FeSO_4_, 0.4 kg B and 60 kg micronutrient per ha) were applied two DAT to all three N treatments at the standard commercial rate of Colombia.

Integrated agronomic practices were adopted to control pests and weeds throughout the trials. Soil samples were collected before and three times during the experiment in each treatment to determine soil type and soil mineral N. At each sampling, fifteen subsamples per treatment were taken at 0–40 cm depth and samples were pooled for each replicate.

### Upland rain‐fed confined field experiment (Santa Rosa, Colombia, 2013–2014)

A field experiment was conducted at CIAT‐Santa Rosa, Colombia, under rain‐fed upland conditions with limited N supply (N_50%_) from November 2013 to March 2014. The trial was planted in a random complete block design with three replicates, with plots measuring 2 × 2 m and 25 × 10 cm plant spacing. Seeds were sown by hand at the rate of 120 kg/ha when soil moisture was about 80% of field capacity. Fertilizer was applied at 90N/70 P/60K kg/ha. The total N application rate of 90 kg/ha (N_50%_) was applied in three splits: 40 kg/ha N as basal, 25 kg/ha N at 40 DAS and 25 kg/ha N at 55 DAS. A dry spell (no rain for a continuous 25 days) coincided with flowering of the plants (Figure S2). Supplemental irrigation was provided whenever severe leaf rolling was apparent. Total rainfall during the cropping period was approximately 146 mm. The soil moisture was monitored throughout the trial using an Aquapro soil moisture device (Aquapro‐Sensors). Plant growth and development was monitored regularly and compared to local check Curinga.

### Trait measurements, chlorophyll and N content

At harvest, seven plants (lowland) or six plants (upland) from each replication per N treatment were sampled for plant height, tiller number, panicle number and length. Samples were oven‐dried at 60 °C for 4 days for dry biomass. Single plant grain weight and 1000GW were adjusted to 12% grain moisture. Field grain yield (kg/ha) was estimated from single plant yield, based on plant density in the plot.

Relative chlorophyll content was determined in fully expanded flag leaves from three stages of crop development using a SPAD‐502 l meter (Minolta). N concentration was measured by Kjeldahl from dried leaf and root samples (hydroponics) and leaf and mature seed (paddy fields) according to Yoshida and Shioya, 1976. Total N content was calculated as N concentration multiplied by dry weight.

### Data Analysis

All statistical analyses were performed using the Statistical Analysis System SAS 9.4 (SAS Institute Inc., Cary, NC, USA). For the lowland field experiments, the mixed model was used, assuming N treatment, genotype and N treatment × genotype effects as fixed with block effect as random. In the presence of significant effect for the model source of variation, means separation procedure was used with Duncan's multiple range test at the level of *P* = 0.05. For estimating the closeness of sample means to the population, we have used standard deviation throughout the data analysis.

The hydroponic and upland rain‐fed experiments’ results were analysed by one‐way ANOVA and compared with those of the WT controls. ANOVA was used to reject the null hypothesis of equal means of transgenic lines and WT controls.

### Metabolite analysis and quantification

Tissue samples for metabolite analysis were collected from rice grown during the 2013 paddy field trial. The penultimate leaf of plants just prior to anthesis and cleaned roots were flash‐frozen in liquid N, lyophilized and ground to a fine powder. Chemicals were 98%+ purity (Alfa Aesar, EMD Millipore, Sigma‐Aldrich). Metabolites and amino acids were extracted using 1 : 1 :0.5 (v : v : v) 0.1 M HCl:methanol:chloroform as described by Hacham *et al*., 2002;. D3‐methionine (CDN Isotopes) was used as an internal standard for all leaf tissue metabolites. D_7_‐alanine (CDN Isotopes) and D_7_‐glucose (Sigma‐Aldrich) were also added to roots prior to extraction as internal standards for the quantification of alanine and sugars, respectively. Extracted metabolites and amino acids were derivatized and analysed by GC/MS (Agilent 6890/5973i) as described by Roessner *et al*., 2000.

Amino acid analysis of leaf and root samples from the hydroponic essay was performed by Quantar (Cali, Colombia, www.quantarlabs.com), following a modified HPLC method.

## Conflict of interest

The authors declare no conflict of interest.

## Authors Contribution

MGS, MI, JvB and JCK designed the research, JvB, MGS, JCK, ZL discussed the data, and MGS, JvB and JCK wrote the manuscript. Rice transformations, plant event advancement and molecular analysis were performed by CD and LW, CD and YL, respectively. Root trait analysis was performed by SO, WS and MGS, and field RT‐PCR expression analyses were performed by MGS and MI. Paddy field and upland rain‐fed confined field trials conducted by MOV and MGS. Statistical analyses were performed by ZL, MGS, MOV and SO.

## Ethical standards

The authors declare that the transgenic experiments comply with the current biosafety laws of the country in which they were performed.

## Supporting information


**Figure S1** Climatic parameters during confined paddy field experiments (August to December, 2012 and February to June, 2013) at CIAT, Palmira. Solar radiation and temperature data are monthly averages and rainfall is monthly total.
**Figure S2** (**a**) Rainfall and temperature pattern during crop period in upland confined rainfed field trial at Santa Rosa, 2013‐2014. (**b**) Time course of the Aquapro soil moisture level at different soil depths (0‐60 cm) during cropping period.

**Table S1** Numerical breakdown of consecutive steps in the development of NERICA‐4 NUE lead events.
**Table S2** Soil physical and chemical properties of confined paddy fields (2012 & 2013) at Palmira and upland rainfed (2014) experiment in Santa Rosa.
**Table S3** Variation in chlorophyll content in flag leaves among transgenic events and controls grown at different N levels during different crop stages in 2012 and 2013 confined paddy field experiments at CIAT, Palmira. Values are means of SPAD measurements of 15 plants ±SD.
**Table S4** Metabolic profiling in penultimate leaves of lead event and WT plants grown during the 2013 lowland confined paddy field trial under different N application rates. Values are means from 3 replications per N rate. LSD_0.05_ is designated for the means across nitrogen rates.
**Table S5** Metabolic profiling in roots of lead event and WT plants grown during the 2013 lowland confined paddy field trial under different N application rates. Values are means from 3 replications per N rate. LSD_0.05_ is designated for the means across nitrogen rates.
**Table S6** Amino acid profiling in flag leaves and roots of lead event and WT plants grown in a hydroponic system under different N application rates. Values are means of 4 plants.
**Table S7** Estimation of agronomic NUE (ANUE) under different N levels at two confined paddy field experiments. GY, grain yield (g/plant); NF, applied nitrogen fertilizer level (kg N/ha). Values are means of three replications.
**Table S8** Primer sequences used in this study.
