# Supplementary material for: Development and field performance of nitrogen use efficient rice lines for Africa
Source: Plant Biotechnol J. 2017 Jan 25;15(6):775–87. doi: 10.1111/pbi.12675 (PMC5425388; doi:10.1111/pbi.12675)
Supplement: Supplementary file 1 — Figure S1 Climatic parameters during confined paddy field experiments (August to December, 2012 and February to June, 2013) at CIAT, Palmira. Solar radiation and temperature data are monthly averages and rainfall is monthly total. Figure S2 (a) Rainfall and temperature pattern during crop period in upland confined rainfed field trial at Santa Rosa, 2013‐2014. (b) Time course of the Aquapro soil moisture level at different soil depths (0‐60 cm) during cropping period. [file PBI-15-775-s001.pdf]

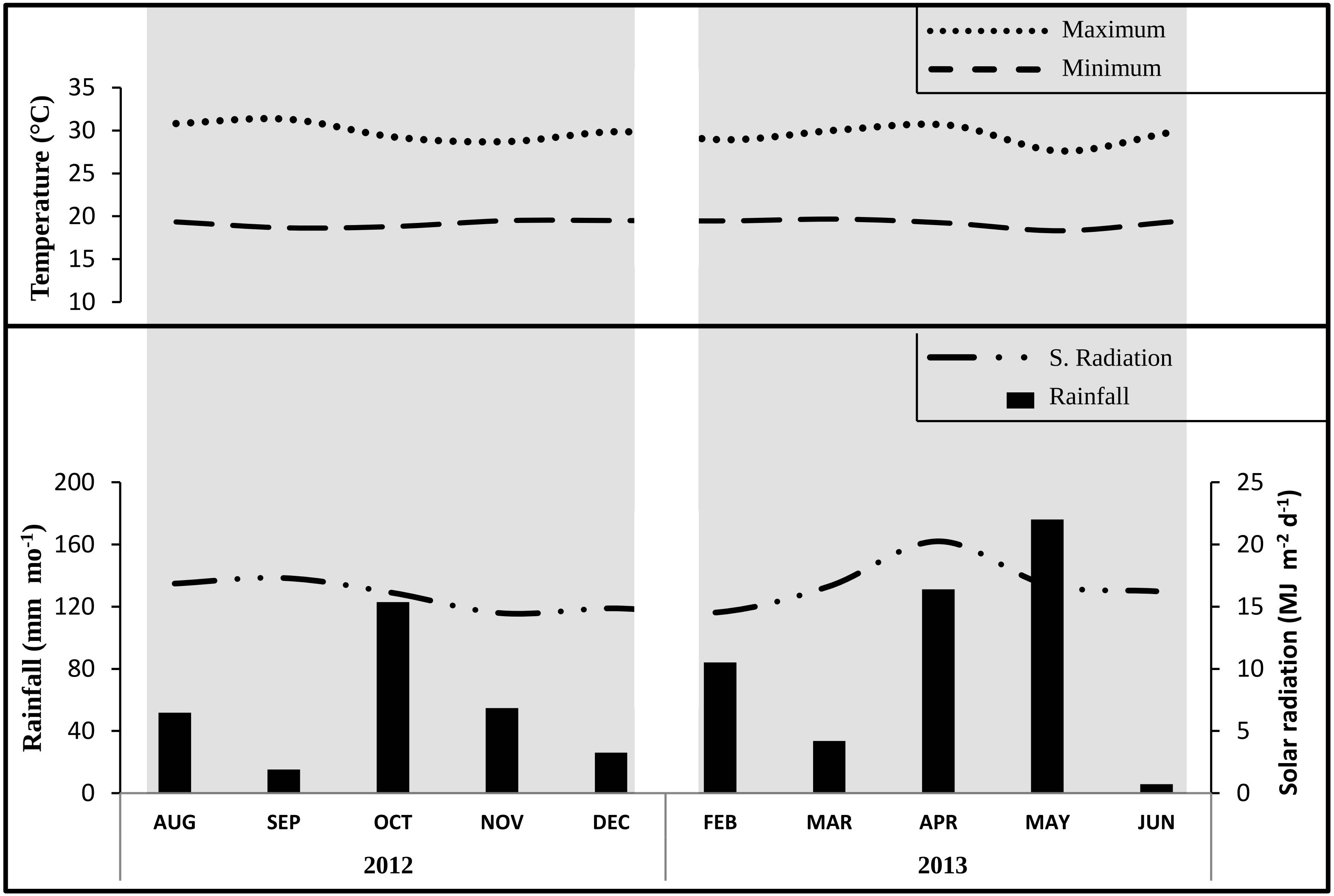

Figure S1. Climatic parameters during confined paddy field experiments (August to December, 2012 and February to June, 2013) at CIAT, Palmira. Solar radiation and temperature data are monthly averages and rainfall is monthly total.

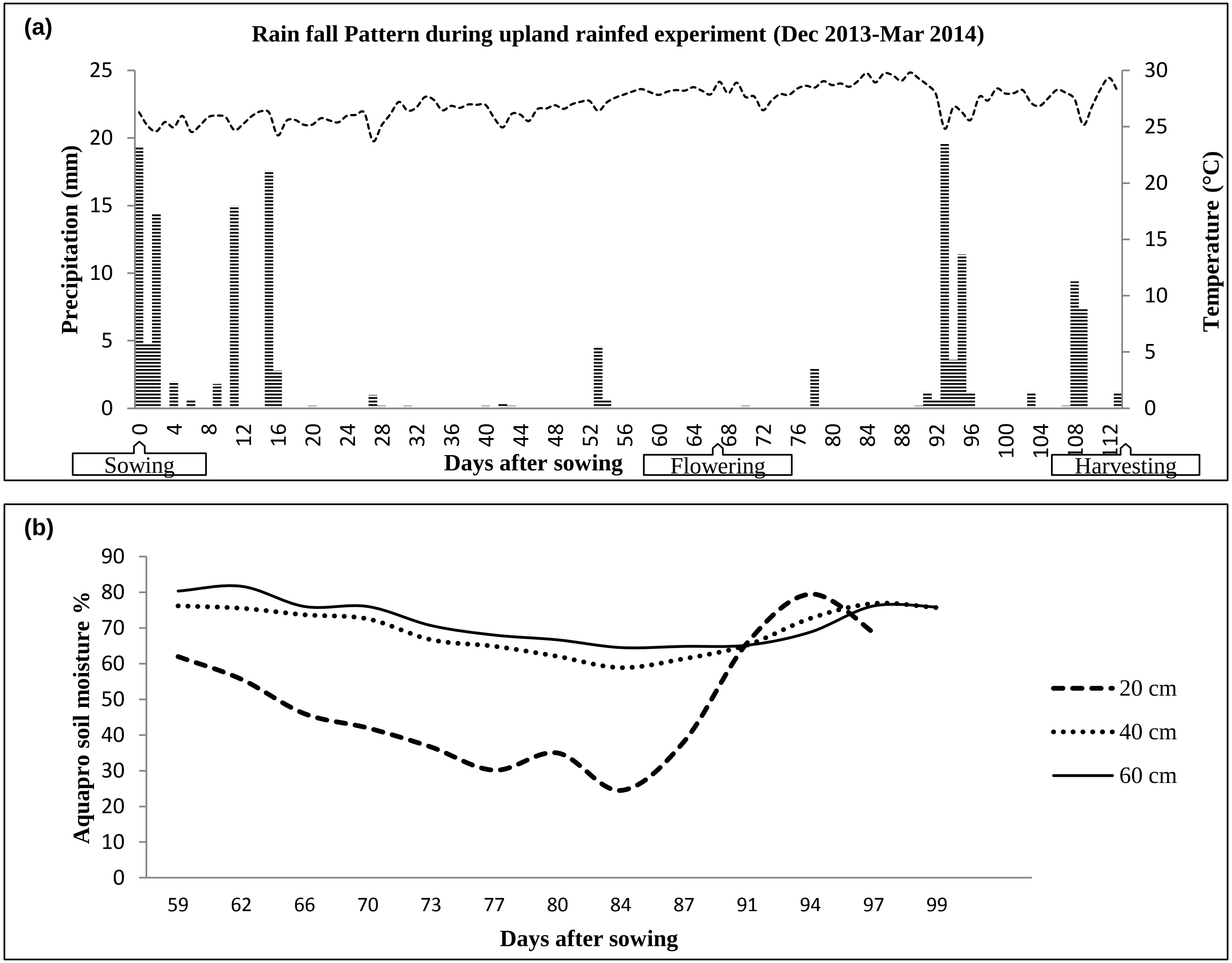

Figure S2. (**a**) Rainfall and temperature pattern during crop period in upland confined rainfed field trial at Santa Rosa, 2013-2014. (**b**) Time course of the Aquapro soil moisture level at different soil depths (0-60 cm) during cropping period.
