# Supplementary material for: Development and field performance of nitrogen use efficient rice lines for Africa
Source: Plant Biotechnol J. 2017 Jan 25;15(6):775–87. doi: 10.1111/pbi.12675 (PMC5425388; doi:10.1111/pbi.12675)
Supplement: Supplementary file 2 — Table S1 Numerical breakdown of consecutive steps in the development of NERICA‐4 NUE lead events. Table S2 Soil physical and chemical properties of confined paddy fields (2012 & 2013) at Palmira and upland rainfed (2014) experiment in Santa Rosa. Table S3 Variation in chlorophyll content in flag leaves among transgenic events and controls grown at different N levels during different crop stages in 2012 and 2013 confined paddy field experiments at CIAT, Palmira. Values are means of SPAD measurements of 15 plants ±SD. Table S4 Metabolic profiling in penultimate leaves of lead event and WT plants grown during the 2013 lowland confined paddy field trial under different N application rates. Values are means from 3 replications per N rate. LSD0.05 is designated for the means across nitrogen rates. Table S5 Metabolic profiling in roots of lead event and WT plants grown during the 2013 lowland confined paddy field trial under different N application rates. Values are means from 3 replications per N rate. LSD0.05 is designated for the means across nitrogen rates. Table S6 Amino acid profiling in flag leaves and roots of lead event and WT plants grown in a hydroponic system under different N application rates. Values are means of 4 plants. Table S7 Estimation of agronomic NUE (ANUE) under different N levels at two confined paddy field experiments. GY, grain yield (g/plant); NF, applied nitrogen fertilizer level (kg N/ha). Values are means of three replications. Table S8 Primer sequences used in this study. [file PBI-15-775-s002.pdf]

Table S1: Breakdown in numbers of consecutive steps in the development of NERICA-4 NUE lead events.

**breakdown**	**pARC321/pPIPRA543 numbers**
Co-cultivated calli	3750
T_0 plants generated on selective medium	365
**transformation efficiency**	**10%**
T_0 plants *nptII+/HvAlaAT+* co-transformed	97
**co-transformation efficiency**	**26.6%**
T_0 plants vector backbone-free	56
**proper T-DNA insertion rate**	**57.7%**
T_0 plants low copy number (≤2) for pARC321 T-DNA	42
**low copy insertion rate**	**75.0%**
T_1 lines with sufficient seed for *nptII* outsegregation analysis	25
T_1 lines with sufficient seed germinating	18
T_1 lines showing outsegregation of pPIPRA543 T-DNA	11
**outsegregation rate**	**61.1%**
T_2 events consisting of only homozygous, marker-free, vector backbone-free, single copy number lines	7

Table S2. Soil physical and chemical properties of confined paddy field (2012 & 2013) at Palmira and upland rainfed (2014) experiment in Santa Rosa.

Soil Chemical and Physical Property	N Treatment			
	Season	0%	50%	100%
Organic Matter (g/kg)	2012	12.87	12.83	13.07
	2013	12.76	13.73	13.13
	2014	-	22.4	-
pH	2012	8.26	8.32	8.3
	2013	7.86	7.81	7.82
	2014		5.2	
P-BrayII (mg/kg)	2012	51.42	32.22	30.66
	2013	38.45	40.19	39.03
	2014	-	7.5	-
NH_4^+ (mg/kg)	2012	2.93	2.95	4.01
	2013	7.79	8.47	11.29
	2014	-	12.60	-
NO_3^- (mg/kg)	2012	0.21	0.42	0.41
	2013	0.62	0.4	0.21
	2014	-	3.10	-
K (cmol/kg)	2012	0.67	0.66	0.7
	2013	0.69	0.7	0.68
	2014	-	0.14	-
CE (ds/m)	2012	0.32	0.29	0.35
	2013	0.33	0.42	0.37
Soil Texture (%)	Clay	Silt	Sand	Texture
Palmira	15.76	65.36	18.88	Silty loam
Santa Rosa	35.93	43.89	20.18	Silty Clay loam

Soil samples were collected before starting the field experiment. Each data point is the average of three soil samples pooled from 15 different sub samples.

Table S3. Variation in chlorophyll content in flag leaves among transgenic events and controls grown at different N levels during different crop stages in 2012 and 2013 confined paddy field experiments at CIAT, Palmira. Values are means of SPAD measurements of 15 plants ±SD.

		2012			2013		
		Developmental stage					
Genotype	Treatment	Flowering	Milky Stage	Dough Stage	Flowering	Milky Stage	Dough Stage
WT	N 0%	42.05 ± 8.20	45.65 ± 1.00	29.43 ± 1.35	46.91 ± 3.43	42.45 ± 3.42	43.19 ± 4.43
	N 50%	48.07 ± 2.62	48.17 ± 2.85	31.67 ± 2.09	49.56 ± 0.53	53.92 ± 0.25	51.61 ± 0.34
	N 100%	50.02 ± 8.16	52.40 ± 2.06	42.26 ± 0.67	52.22 ± 1.30	72.78 ± 0.94	53.69 ± 2.61
NUE-1	N 0%	42.65 ± 8.97	44.10 ± 1.25	28.90 ± 1.52	*50.17 ± 1.82	*45.93 ± 0.59	*47.70 ± 0.53
	N 50%	42.53 ± 6.61	49.47 ± 2.28	33.48 ± 1.15	52.14 ± 1.53	55.89 ± 1.05	53.75 ± 1.32
	N 100%	50.56 ± 4.78	51.18 ± 1.97	40.00 ± 1.84	54.04 ± 0.86	70.58 ± 1.25	55.20 ± 0.87
NUE-2	N 0%	42.15 ± 8.02	†42.57 ± 0.31	29.23 ± 0.49	47.71 ± 3.33	*45.29 ± 1.12	45.80 ± 2.61
	N 50%	48.93 ± 5.34	48.89 ± 0.50	33.55 ± 1.89	51.06 ± 1.49	*57.87 ± 0.83	52.45 ± 0.93
	N 100%	51.68 ± 2.59	51.57 ± 1.43	†39.18 ± 0.16	*56.44 ± 1.92	*77.51 ± 2.27	*60.29 ± 6.66
NUE-2N	N 0%	-	-	-	46.30 ± 1.09	44.00 ± 0.91	42.89 ± 0.79
	N 50%	-	-	-	50.33 ± 1.41	*57.82 ± 2.61	51.53 ± 1.00
	N 100%	-	-	-	53.30 ± 1.48	74.81 ± 2.93	54.59 ± 1.23
NUE-3	N 0%	43.18 ± 2.49	47.30 ± 0.92	31.24 ± 1.90	46.75 ± 1.92	*48.58 ± 1.68	*48.79 ± 2.06
	N 50%	48.59 ± 2.42	*51.53 ± 1.47	*36.48 ± 1.73	51.85 ± 1.78	*62.79 ± 5.97	53.09 ± 1.42
	N 100%	49.70 ± 1.04	54.65 ± 1.65	44.45 ± 1.46	54.39 ± 1.02	72.15 ± 2.08	53.71 ± 0.72
NUE-4	N 0%	45.88 ± 2.24	*49.27 ± 0.28	*34.49 ± 3.39	-	-	-
	N 50%	49.02 ± 0.67	49.56 ± 1.17	*36.30 ± 2.41	-	-	-
	N 100%	53.13 ± 0.16	52.35 ± 0.31	44.49 ± 1.52	-	-	-
NUE-5	N 0%	45.35 ± 4.93	45.71 ± 0.52	*34.82 ± 2.17	-	-	-
	N 50%	48.08 ± 2.07	46.51 ± 1.75	*38.60 ± 2.65	-	-	-
	N 100%	48.82 ± 6.76	50.16 ± 2.74	43.59 ± 1.04	-	-	-
NUE-6	N 0%	*48.41 ± 1.18	*48.15 ± 2.85	*34.23 ± 2.17	49.66 ± 2.87	*50.01 ± 4.01	*47.65 ± 3.46
	N 50%	48.30 ± 2.94	*50.99 ± 0.45	*36.46 ± 1.83	*52.83 ± 0.74	*61.94 ± 3.82	53.22 ± 0.39
	N 100%	48.89 ± 7.46	*55.95 ± 1.25	*46.14 ± 1.18	53.67 ± 1.93	†66.93 ± 0.70	54.03 ± 1.06
NUE-6N	N 0%	-	-	-	49.29 ± 1.15	*48.06 ± 1.26	*48.17 ± 3.39
	N 50%	-	-	-	52.01 ± 0.96	54.49 ± 0.66	51.39 ± 1.40
	N 100%	-	-	-	*55.83 ± 2.36	72.72 ± 2.87	55.43 ± 2.44
Curinga	N 0%	46.05 ± 3.58	40.72 ± 1.79	31.16 ± 1.13	42.29 ± 2.33	42.25 ± 0.94	41.63 ± 1.85
	N 50%	44.11 ± 6.05	45.58 ± 0.45	33.00 ± 2.23	46.05 ± 0.60	49.76 ± 0.70	45.89 ± 1.00
	N 100%	50.75 ± 1.69	47.93 ± 1.11	37.20 ± 1.20	49.08 ± 1.05	49.88 ± 0.39	46.77 ± 0.98
Fedearroz 174	N 0%	45.73 ± 0.84	31.20 ± 1.00	26.46 ± 0.87	34.27 ± 1.71	32.37 ± 0.70	29.94 ± 0.69
	N 50%	45.97 ± 9.01	33.11 ± 2.37	31.23 ± 0.67	42.86 ± 0.24	39.18 ± 1.52	33.73 ± 1.36
	N 100%	49.71 ± 4.17	36.51 ± 1.10	34.79 ± 0.15	44.63 ± 1.54	43.53 ± 1.75	38.09 ± 1.76
N Level (N)		**	**	**	**	**	**
Genotype (G)		ns	**	**	**	**	**
N x G		ns	*	**	*	**	**
CV%		11.2	3.1	4.6	3.3	3.9	4.4

* = significantly higher than WT p<0.05 ** = significantly higher than WT at p<0.01 ns=not significant p>0.05

† = significantly lower than WT at p<0.05

Table S4. Metabolic profiling in penultimate leaves of lead event and WT plants grown during the 2013 lowland confined paddy field trial under different N application rates. Values are means from 3 replications per N rate. $LSD_{0.05}$ is designated for the means across nitrogen rates.

Metabolites (μg/g DW tissue)	Field nitrogen rate									Mean across nitrogen rates			
	N0%			N50%			N100%						
	N2	N6	WT	N2	N6	WT	N2	N6	WT	$LSD_{0.05}$	N2	N6	WT
Oxalate	146	123	162	174	184	135	159	138	175	32	160	148	157
GABA	228	197	231	165	181	160	209	192	181	81	201	190	191
Phosphoric Acid	943	1048	1402	841	860	734	740	800	680	316	841	903	939
Fumarate	76	72	77	91	106	80	131	118	100	95	99	98	86
Pyruvate	168	191	206	102	136	121	105	113	111	59	125	147	146
a-Ketoglutarate	296	699	525	238	426	339	296	370	198	292	276	498	354
Shikimate	450	272	321	*811	351	258	351	197	474	192	537	274	351
Citrate	1214	2452	1645	1157	1605	1568	1370	1336	982	831	1247	1798	1398
Isocitrate	115	1478	364	85	110	132	73	84	70	611	91	557	189
Glycerate	498	432	455	468	448	389	311	392	453	114	426	424	432
Tyramine	7	†4	16	6	4	7	3	6	5	4	†6	†5	9
Malate	2227	3801	2756	1916	2465	2710	2139	2130	1634	1360	2094	2799	2367
Arabinose	1880	1803	1660	1350	1320	1306	1316	1253	1460	618	1515	1459	1476
Ribose	76	83	79	57	42	59	66	65	66	31	66	63	68
Fructose	51897	45735	56131	30640	32424	40115	30050	36638	30224	16093	37529	38266	42157
Glucose	60295	65567	68282	43336	47680	46298	40582	46167	38207	25391	48071	53138	50929
Fructose-6-PO4	42	41	41	58	59	54	50	46	48	14	50	49	48
Glucose-6- PO4	25	26	28	38	28	21	21	13	20	12	28	22	23

Amino acids (μg/g DW tissue)	Field Nitrogen Rate									Mean across nitrogen rates			
	N0%			N50%			N100%						
	N2	N6	WT	N2	N6	WT	N2	N6	WT	$LSD_{0.05}$	N2	N6	WT
Glx	1192	955	1099	1119	1272	1152	1239	1379	1226	291.6	1184	1202	1159
Aspartic acid	104	65	88	158	134	126	181	148	163	31.4	148	116	126
Threonine	15	8	9	22	19	16	32	21	23	9.5	23	16	16
Isoleucine	4	4	3	6	4	5	7	4	8	2.3	6	4	5
Methionine	8	7	5	7	7	7	11	9	11	2.1	9	8	8
Serine	13	13	6	22	13	11	38	16	22	12.7	24	14	13
Glycine	*50	26	22	19	*28	18	31	27	40	8.2	33	27	26
Tyrosine	63	42	39	42	34	38	22	19	17	10.1	*42	32	31
Phenylalanine	12	16	15	16	15	15	19	12	15	3	15	14	15
Alanine	668	452	414	381	568	422	488	538	576	219.6	513	519	470
Leucine	3	4	2	7	3	4	6	3	8	3.1	6	3	5
Valine	*27	18	16	*24	20	15	24	23	24	4.6	*25	20	18

Levels of His, Pro, Asn, Lys and Trehalose were below the level of quantification; Arg, Cys and Trp were not measured in this analysis. Glx is the sum of glutamine and glutamate.

***** = significantly higher than WT at $p<0.05$; **†** = significantly lower than WT at $p<0.05$. LSD0.05 = least significant difference at $p<0.05$ to detect significance in event-WT pairs across all N rates.

Table S5. Metabolic profiling in roots of lead event and WT plants grown during the 2013 lowland confined paddy field trial under different N application rates. Values are means from 3 replications per N rate. LSD$_{0.05}$ is designated for the means across nitrogen rates.

Metabolites (µg/g DW tissue)	Field Nitrogen Rate									Mean across nitrogen rates			
	N0%			N50%			N100%						
	N2	N6	WT	N2	N6	WT	N2	N6	WT	LSD$_{0.05}$	N2	N6	WT
Oxalate	†51	143	172	374	79	186	135	172	106	93	167	137	153
GABA	*367	209	168	180	211	115	215	176	226	92	271	195	179
Phosphoric Acid	*163	52	48	77	92	40	77	104	104	63	114	86	65
Fumarate	236	†85	292	131	91	114	†58	†86	181	96	155	†87	226
Pyruvate	77	69	81	90	77	63	74	83	74	28	80	77	76
a-Ketoglutarate	7	6	9.3	10	6	5.2	9	12	4.3	4.2	8	9	6.9
Shikimate	*342	*273	165	292	*325	137	*321	*252	179	64	*322	*279	165
Citrate	7017	7125	5712	6672	7054	4687	5713	6501	5043	1060	*6546	*6837	5318
Isocitrate	51	40	37	32	*56	28	*56	47	42	11	47	48	37
Glycerate	*67	*57	24	40	*62	19	*52	*52	33	12	*55	*56	26
Malate	*45125	39148	30126	39881	43520	21083	*39699	*41946	19872	8377	*42076	*41596	25201
Arabinose	*697	*702	375	634	621	430	988	517	425	239	*762	600	401
Ribose	*58	*69	22	41	*60	29	24	38	40	13	*43	*53	29
Trehalose	7	5	7	11	9	6	9	8	3	5	9	7	5
Glucose	41319	†31232	39090	56318	20140	44781	24837	37139	36055	10700	40895	30594	39027
Fructose	*27883	23748	21399	30909	24710	23795	*29107	*22657	21035	2572	*29097	23555	21677
Fructose-6-PO4	42	36	10	39	4	9	10	8	29	34	32	15	16
Glucose-6-PO4	6	4	4	4	†3	6	3	7	3	7	5	5	4

Amino Acids (µg/g DW tissue)	Field Nitrogen Rate									Mean across nitrogen rates			
	N0%			N50%			N100%						
	N2	N6	WT	N2	N6	WT	N2	N6	WT	LSD$_{0.05}$	N2	N6	WT
Glx	*646	*624	278	449	679	346	*1002	701	647	204	*692	*673	412
Histidine	8	†6	13	9	12	2	†4	†6	8	6	7	8	9
Proline	*15	9	2	3	3	3	8	6	7	4	*10	6	4
Aspartic acid	*139	75	68	101	77	87	179	150	145	32	*140	108	97
Asparagine	*27	*26	14	20	24	11	19	12	13	7	*23	19	13
Threonine	*37	*30	19	34	35	29	*61	48	34	9	*43	*39	26
Isoleucine	*49	28	13	23	18	16	25	27	25	11	*35	25	18
Methionine	14	16	13	17	18	13	21	17	18	5	17	17	15
Lysine	249	205	225	338	253	231	18	103	17	205	208	175	157
Serine	*110	68	45	63	72	67	*190	103	113	21	*120	84	71
Glycine	*37	*17	9	9	*26	8	*33	19	19	4	*28	*21	12
Tyrosine	23	17	21	†14	†12	21	19	17	14	9	19	16	19
Phenylalanine	5	2	3	4	2	4	6	10	10	3	5	6	6
Alanine	*208	*137	87	119	*209	121	*342	†182	249	13	*221	*177	147
Leucine	*35	23	9	14	8	11	14	13	15	8	*23	15	11
Valine	*36	*50	14	28	29	22	61	32	40	14	*41	36	24

Levels of Tyramine were below the level of quantification; Arg, Cys and Trp were not measured in this analysis. Glx is the sum of glutamine and glutamate. = significantly higher than WT at $p<0.05$; † = significantly lower than WT at $p<0.05$. LSD0.05 = least significant difference at $p<0.05$ to detect significance in event-WT pairs across all N rates.

Table S6. Amino acid profiling in flag leaves and roots of lead event and WT plants grown in a hydroponic system under different N application rates. Values are means of 4 plants.

Amino acids (µg/g DW tissue)	Leaf						Root					
	50 µM NH4+			500 µM NH4+			50 µM NH4+			500 µM NH4+		
	N2	N6	WT	N2	N6	WT	N2	N6	WT	N2	N6	WT
Glutamatic acid	48	103	60	138	123	119	355	467	487	156	208	98
Histidine	9	27	10	118	102	70	64	58	98	41	44	35
Proline	ND	ND	2	6	5	5	24	27	24	12	13	8
Aspartic acid	43	25	17	47	14	21	74	59	227	106	52	58
Asparagine	ND	3	2	255	132	52	130	118	106	166	144	166
Threonine	13	35	14	48	56	51	43	53	74	36	39	28
Isoleucine	52	52	39	28	37	44	101	106	92	71	72	55
Methionine	60	50	42	21	32	31	95	143	88	40	40	44
Lysine	12	23	69	26	17	36	127	155	134	61	58	50
Serine	45	72	63	32	93	86	247	291	312	172	142	135
Glycine	25	22	27	*150	*153	110	*94	37	27	36	36	26
Cysteine	ND	25	ND	ND	ND	ND	16	16	37	19	14	9
Tryptophan	26	33	16	14	6	19	23	26	22	14	15	8
Tyrosine	15	79	18	41	64	42	ND	16	ND	19	20	3
Phenylalanine	11	19	10	10	11	11	42	50	46	27	28	23
Alanine	44	56	42	*281	*280	192	*83	*102	51	61	61	34
Leucine	15	22	17	16	17	16	59	59	66	34	39	32
Valine	131	109	79	46	103	65	46	51	62	39	42	31

All levels of Gln and Arg were below the level of quantification; in some cases, Pro, Cys and Tyr were not detected (ND) in this analysis. ***** = significantly higher than WT at $p<0.05$

Table S7. Estimation of agronomic NUE (ANUE) under different N levels at two confined paddy field experiments. GY, grain yield (g/plant); NF, applied nitrogen fertilizer level (kg N/ha). Values are means of three replications.

	**ANUE = $(GY_{100\%} - GY_{0\%})/NF_{100\%}$**		**ANUE = $(GY_{50\%} - GY_{0\%})/NF_{50\%}$**	
**Event**	**2012**	**2013**	**2012**	**2013**
NUE-1	18.4±2.19	†27.58±2.31	†11.31±4.07	33.88±3.43
NUE-2	27.12±1.39	*42.01±0.55	*36.35±0.96	*54.22±2.55
NUE-2N	-	33.35±0.16	-	44.22±7.53
NUE-3	22.02±0.19	33.12±3.69	21.07±1.91	43.46±7.30
NUE-4	15.53±1.77	-	†10.54±1.09	-
NUE-5	14.6±1.66	-	†13.16±1.64	-
NUE-6	20.05±4.39	40.24±2.15	†9.07±1.83	*58.12±0.58
NUE-6N	-	33.76±3.01	-	37.85±4.87
WT	20.59±0.96	34.58±0.90	25.2±4.10	42.61±1.63

$NF_{100\%}$= 180 kg N/Ha; $NF_{50\%}$= 90 kg N/Ha; *significantly higher than WT at p<0.05; † = significantly lower than WT at p<0.05.

Table S8. Primer sequences used in this study

Experiment	Primer name	Primer sequence (5' - 3')
Conventional PCR	HvAlaAT Fw1	CGCAGAAGGAACCCGTCTTCTA
	HvAlaAT Rv1	GAGATTGTGGGTTCCCAATGTTAC
	Nptll Fw1	TATCACGGGTAGCCAACGCTATGT
	Nptll Rv1	ACAAGATGGATTGCACGCAGGTTC
	OsADH Fw	CGGGGATGGTTCATCTAGCC
	OsADH Rv	CCAGCAACCAGAGCAGCAG
qPCR for copy number analysis	HvAlaAT Fw2	ATGGCTGCCACCGTCGCCGTGGAC
	HvAlaAT Rv2	GAGATTGTGGGTTCCCAATGTTAC
	OsUbi5 Fw	ACCACTTCGACCGCCACTACT
	OsUbi Rv	ACGCCTAAGCCTGCTGGTT
	NptII Fw2	AAGATGGATTGCACGCAGGTT
	NptII Rv2	AGAGCAGCCGATTGTCTGTTG
	OsAct Fw	CCTCTTCCAGCCTTCCTTCATA
	OsAct Rv	GCAATGCCAGGGAACATAGTG
RT-PCR for RNA	HvAlaAT Fw	GTATGCTGTGCGTGGAGAGA
	Nos Rv	TGCCAAATGTTTGAACGATC
	18S rRNA_Fw	ATGATAACTCGACGGATCGC
	18S rRNA_Rv	CTTGGATGTGGTAGCCGTTT
